# Mosquito Feeding Habits in Coastal French Guiana: Mammals in the Crosshairs?

**DOI:** 10.3390/insects15090718

**Published:** 2024-09-19

**Authors:** Amandine Guidez, Sourakhata Tirera, Stanislas Talaga, Guillaume Lacour, Romuald Carinci, Edith Darcissac, Damien Donato, Pascal Gaborit, Emmanuelle Clervil, Yanouk Epelboin, Benoit de Thoisy, Isabelle Dusfour, Jean-Bernard Duchemin, Anne Lavergne

**Affiliations:** 1 Unité d‘Entomologie Médicale, Institut Pasteur de la Guyane, 97354 Cayenne, France; stalaga@pasteur-cayenne.fr (S.T.); glacour.84@gmail.com (G.L.); rcarinci@pasteur-cayenne.fr (R.C.); pgaborit@pasteur-cayenne.fr (P.G.); eclervil@pasteur-cayenne.fr (E.C.); idusfour@gmail.com (I.D.); jean-bernard.duchemin@pasteur.fr (J.-B.D.); 2 Laboratoire des Interactions Virus-Hôtes, Institut Pasteur de la Guyane, 97354 Cayenne, France; stirera@pasteur-cayenne.fr (S.T.); edarcissac@pasteur-cayenne.fr (E.D.); ddonato@pasteur-cayenne.fr (D.D.); bdethoisy@pasteur-cayenne.fr (B.d.T.); anne.lavergne@pasteur.fr (A.L.); 3 Microbiota of Insect Vectors Group, Institut Pasteur de la Guyane, 97354 Cayenne, France; yepeboin@pasteur-cayenne.fr

**Keywords:** mosquito feeding habits, blood-fed females, host associations, French Guiana

## Abstract

**Simple Summary:**

Mosquitoes transmit pathogens through cycles involving animals and humans. Understanding which animals mosquitoes feed on helps us to monitor these diseases. In our study, we examined blood-fed female mosquitoes from various sites in French Guiana to determine their feeding preferences. We used DNA (genetic material) present in their blood meals to identify the animals they had fed on. We discovered that most mosquitoes we studied, especially those from the *Culex* group, prefer feeding on mammals like humans and other animals, followed by birds, amphibians, and reptiles. We identified 46 different animal species in the blood meals. Our findings provide important information on mosquito feeding habits and help improve the tracking of diseases spread by mosquitoes, which is crucial for public health.

**Abstract:**

Pathogens transmitted by mosquitoes (Diptera, Culicidae) in sylvatic or urban cycles involve wild or domestic animals and humans, driven by various mosquito species with distinct host preferences. Understanding mosquito–host associations is crucial for ecological insights and pathogen surveillance. In this study, we analyzed mosquito blood meals from coastal French Guiana by amplifying and sequencing host DNA from blood-fed females. Using the 12S ribosomal RNA gene and Sanger sequencing, we identified blood meals from 26 mosquito species across six genera, with 59% belonging to the *Culex* genus. Nanopore sequencing of selected samples showed 12 mosquito species with one to three mixed blood-meal sources. Mammals were the primary hosts (88%), followed by birds (7%), squamates (3%), and amphibians (2%), indicating a strong preference for mammalian hosts. A total of 46 vertebrate host species were identified, demonstrating high host diversity. This research provides insights into mosquito host usage and highlights the complexities of monitoring arboviruses of public health concern.

## 1. Introduction

A total of 3726 species of mosquito (family Culicidae, order Diptera) are currently formally recognized globally [1], including less than 400 species known to be vectors of pathogens (virus, bacteria, or parasites) [2], meaning they are able to transmit infectious pathogens between humans, animals or from animals to humans. The transmission of pathogens mainly occurs when mosquitoes feed on an infected host (human or animal) during a blood meal and subsequently transmit them to another vertebrate during a new blood feed, once the pathogen has replicated or multiplied. Arboviruses, protozoa, and filariae ingested with blood into a susceptible mosquito will first develop within the mosquito’s digestive tract, and after a lapse of time, migrate to the salivary glands [3] or mouthparts of the vector [4]. Mosquitoes can subsequently infect multiple vertebrate host species during blood feeding. Nevertheless, not all vertebrate hosts develop pathogen loads that are high enough to infect the mosquitoes that feed on them [5]. This inter-relationship between mosquitoes, hosts, and pathogens is often complex. Consequently, understanding the host associations with mosquitoes is a crucial step in studying these dynamics [6].

Hematophagous female mosquitoes have the ability to feed on a wide range of vertebrates, including mammals, birds, reptiles, amphibians, and even fishes [7,8,9]. This spectrum encompasses both warm-blooded animals, such as mammals and birds, and cold-blooded animals, like snakes and frogs. Some mosquito species can also feed on insects, including nymphal cicadas, lepidopterous larvae, and mantids [10], and *Uranotaenia sapphirina* feed on earthworms and leeches [11] in eastern North America. According to their evolution and life-history traits, mosquito species have each developed their own blood-feeding behavior. Many mosquito species demonstrate a broad range of feeding habits: generalist feeders often display opportunistic behavior, feeding on whatever they can, whereas others exhibit stricter and consistent preferences, either favoring certain classes of hosts (such as mammals or birds) or specific species (such as human). For example, *Culex erraticus*, a bridge vector of eastern equine encephalitis virus (EEEV) and a potential bridge vector of West Nile virus (WNV) in the United States, considered an opportunistic feeder, seems to preferentially target certain avian species for their blood meal [12,13]. *Aedes aegypti*, one of the most medically important vectors worldwide, notably causing dengue, yellow fever, chikungunya, or Zika viral infections, is considered highly anthropophilic [14,15,16].

Female mosquitoes ingest and digest blood to produce viable eggs during a gonotrophic cycle [17]. Interruptions during feeding can lead a mosquito to harvest more than one blood meal before entering the resting and digestion phase of their gonotrophic cycle with a complete meal. For example, the occurrence of multiple blood meals within a single gonotrophic cycle is frequently observed in *Anopheles* species [18,19], indicating a common feeding behavior among these species. Multiple blood feeding can have implications for the transmission of arthropod-borne diseases, as it means that an infectious mosquito that feeds successively on two susceptible hosts could effectively transmit pathogens to both. This scenario increases the likelihood of multiple transmission events occurring in a single gonotrophic cycle and therefore has an impact on the vector control strategy or/and dynamics of mathematical models [20]. In South America, the genus *Anopheles* is the most widely studied [2], as several species of the genus are implicated in malaria parasite transmission, but a notable lack of research on the feeding habits of the majority of the other mosquito genera has been underlined recently [2]. In French Guiana, mosquito–host relationships are largely understudied. However, this French overseas department has diverse ecological zones, with a rich biodiversity of vertebrate host species, and over 245 currently described mosquito species, harboring one of the highest relative species densities of mosquitoes anywhere in the world [21,22,23] Interactions between all these populations across different zones can be a source of pathogen maintenance or the emergence of mosquito-borne pathogens, underscoring mosquito–host feeding preferences as a crucial parameter.

This study aims to enhance the understanding of mosquito–host relationships by utilizing a collection of blood-fed females sampled along the coast of French Guiana and most likely to interact with humans. Using molecular approaches, an investigation was conducted to identify collected females at the species level, to determine the sources of their blood meals, to investigate the detection of multiple blood meals, and to contribute to understanding the blood-feeding patterns of mosquitoes in French Guiana. The broader goal is to enhance knowledge about potential neotropical vectors and their role in disease transmission cycles.

## 2. Materials and Methods

### 2.1. Field Collection of Blood-Fed Female Mosquitoes 

**Figure 1:**
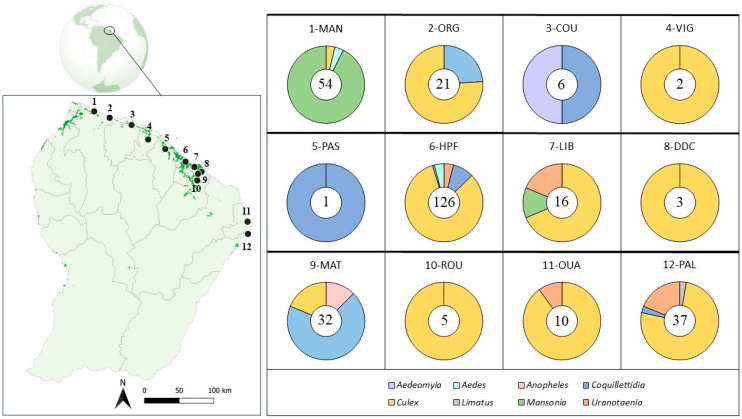
Localization of the 12 sampling sites in French Guiana, along with the genus proportion of blood-fed mosquitoes (depicted as pie charts) and the numbers of blood-fed females for each site (indicated in the middle of the chart). The sites, arranged from west to east, are named as follows: Mana (1-MAN), Organabo (2-ORG), Counamama (3-COU), Viguié (4-VIG), Passoura (5-PAS), Hameau Préfontaine (6-HPF), Pointe Liberté (7-LIB), Dégrad des Cannes (8-DDC), Matoury (9-MAT), Roura (10-ROU), Ouanary (11-OUA), and Trois Palétuviers (12-PAL). Urban fabric coverage is indicated in deep green on the map.

A collection of blood-fed female mosquitoes was established from field sampling performed from 2018 to 2023 at 12 sites along the coast of French Guiana (Figure 1). Mosquito collections were achieved either passively by using traps (CDC UV/light or mosquito magnet) overnight or actively using hand-held aspirators (CDC backpack aspirator or Prokopack aspirator) in supposed resting sites or resting boxes (an artificial box placed in strategic locations and designed to attract resting mosquitoes). Details of trap types and collection conditions for each site are provided as supplementary data (Table S1). Most of selected sites are located in peri-urban areas (with less than 40% urban coverage, except for site 9-MAT) and are situated close to forests (within 0–468 m, except for sites 1-MAN and 8-DDC, which are more than 1000 m). These sites cover a range of habitats typical of the coastal regions of French Guiana surrounding urban areas. Most of the blood-fed females were found in a semi-natural forest area (6-HPF), a coastal area (7-LIB), a port area (8-DDC), a forest area (10-ROU), and areas surrounding Ouanary (11-OUA) in an estuary zone and the river bordering Trois Palétuviers (12-PAL) municipalities. The sampled sites also include a rice-field rural zone in Mana (1-MAN), an urban area in Matoury (9-MAT), and four coastal savannah areas: Organabo (2-ORG), Counamama (3-COU), Viguié (4-VIG), and Passoura (5-PAS) [24]. After collection, mosquito specimens were morphologically identified to species level when possible and kept frozen at −80 °C.

### 2.2. DNA Extraction and PCR Amplification

For nucleic acid isolation, each individual mosquito specimen was homogenized by bead beating (Precellys Evolution; Bertin Technologies, Paris, France) at 7000 rpm for 1 min. This was carried out twice, with a 30 s interval, in a 2 mL tube with 500 μL of cell culture medium (Dulbecco’s Modified Eagle Medium; Sigma-Aldrich, St. Louis, MO, USA). DNA was extracted from 200 μL of the homogenate using the NucliSENS easyMAG^®^ bio-robot (bioMérieux, Durham, NC, USA) following the manufacturer’s protocol.

Due to the challenge of the difficult morphological identification of field-caught females of the *Culex* genus, which constituted the majority of specimens in this study, a molecular approach was used for species identification. Amplification by polymerase chain reaction (PCR) was conducted on the 658 bp DNA barcode fragment from the mitochondrial cytochrome oxidase I (COI) gene using the primer pair LCO-1490/HCO-2198 following protocols previously described [25,26]. PCR products were visualized on 1% agarose gels, containing Midori Green Advance (Biozym Biotech, Hessisch Oldendorf, Germany), or analyzed through capillary electrophoresis using the QIAxcel Advanced BioAnalyzer (Qiagen GmbH, Hilden, Germany).

To identify the vertebrate origin of each blood meal, a short mitochondrial 12S ribosomal RNA (12S rRNA) gene [27] was studied. This marker has already been utilized for the identification of Amazonian vertebrates in dipteran and has an accessible reference library for species found in French Guiana [28]. PCR amplification was performed in 25 μL mixtures containing 2 μL of the DNA template, 0.2 μL of AmpliTaq Gold^®^ (5 U μL^−1^, Applied Biosystems, Foster City, CA, USA), 2.5 μL of ×PCR buffer (provided with Amplitaq Gold^®^, Applied Biosystems, Carlsbad, CA, USA), 0.5 μL of dNTPs (2.5 mM each, Promega, Madison, WI, USA), 1 μL of each primer (10 μM) (12S-V5-forward: 5′-TAGAACAGGCTCCTCTAG-3′, 12S-V5-reverse: 5′-TTAGATACCCCACTATGC-3′), 2.5 μL of MgCl2 (25 mM, Applied Biosystems), and nuclease-free water (Ambion™, Austin, TX, USA), as described previously [27,28].

### 2.3. Identification by Sanger Sequencing

Successful amplifications for mosquito and blood-meal identification were sequenced using the primers used for amplification in the Genewiz sequencing service (Genewiz from Azenta Life Sciences, Liepzig, Germany). Sequences were edited using the Molecular Evolutionary Genetics Analysis (MEGA) X software [29], assembled, and aligned with the Clustal ω2 algorithm using the default parameters. Each specimen was then assigned to a mosquito species by using the Nucleotide Basic Local Alignment Search Tool (BLASTn) to compare each sequence to sequences accessible in GenBank^®^ (www.ncbi.nlm.nih.gov/genbank/, accessed on 10 May 2024) and/or in the Barcode of Life Data Systems (BOLD) [30]. The identification of mosquito species was carried out for all specimens. The percentage identity and query coverage parameter was extracted for each specimen. Identification of species based on the best close match using GenBank^®^ or BOLD sometimes returned unsuccessfully with a low percentage of identity. To avoid misidentification, we therefore only analyzed specimens with an identification to the species level with a best scoring match and match value up to 97.5% nucleotide identity. Host sequences were considered reliable if the percentage of nucleotide identity with a GenBank entry was also higher than 97.5% and if the host species identified was known to be present in French Guiana.

### 2.4. Library Preparation and Nanopore MinION Sequencing

We referred to Oxford Nanopore Technologies (ONT; Oxford, UK) and used the MinION device for sequencing a selected subset of samples to detect multiple blood-meal hosts. The same PCR products used for Sanger sequencing, targeting the 12S gene for blood-meal identification, were used to produce the libraries for MinION sequencing according to the base protocol from Oxford Nanopore (Ip CLC, Loose M, Tyson JR et al. MinION Analysis and Reference Consortium: Phase 1 data release and analysis. F1000Research 2015;4:1075) and the ARTIC nCoV-2019 sequencing protocol (https://www.protocols.io/view/ncov-2019-sequencing-protocol-v2-bdp7i5rn, accessed on 28 March 2022). Briefly, amplicons DNA ends were repaired and dA-tailed using the NEBNext End Repair/dA-tailing module (NEB #E7546). Then, based on the ligation sequencing kit (SQK-LSK109, Oxford Nanopore Technologies plc, Oxford, UK), a unique dT-tailed barcode adapter was ligated on the dA-tailed template using the ONT kit EXP-NBD104. Barcoded samples were then pooled together. After clean-up with 0.4× *Agencourt* AMPure beads XP (Beckman Coulter, Brea, CA, USA) to remove contaminants from the sample, the DNA concentration was assessed using the Qubit dsDNA HR Assay (Life Technologies, Grand Island, NY, USA). Sequencing adapters were ligated to barcoded DNA using the *NEBNext^®^ Quick Ligation Module* (NEB, # E6056S, New England Biolabs, Inc., Ipswich, MA, USA). The ligation mixture was incubated at 20 °C for 15 min and purified using 1X *Agencourt* AMPure beads XP. The final library was quantified using a Qubit^®^ dsDNA HS Assay (Life Technologies, Grand Island, NY, USA) and the 20 ng sequencing library was loaded onto MinION flowcells (FLO-MIN106D, Nanopore Oxford, Oxford, UK).

### 2.5. Bioinformatics Analyses

Once the sequencing was completed, we used guppyplex from the artic pipeline (https://github.com/artic-network/fieldbioinformatics, accessed on 9 August 2024) (version 1.2.1) to aggregate pre-demultiplexed reads for each barcode. Read length ranges were set at a minimum of 50 and maximum of 300 for 12S sequences; the minimum quality was set at eight. In order to generate consensus for each barcode, NGSpeciesID [31] was used with parameters set (--medaka–abundance_ratio 0.01–rc_identity_threshold 0.95). The corresponding primers were given as the input and all other parameters were set to default. The d-chimer program (https://github.com/stirera/d-chimer_v1 accessed on 9 August 2024) was used to perform a homology search against a merged NCBI and BOLD nucleotide database to assign consensuses to reference sequences and obtain taxonomic information. The best scoring matches were chosen for each consensus sequence generated. Whenever necessary (multiple equal matches), the consensus sequence was assigned to the Lowest Common Ancestor (LCA) using an in-house Python script. At the end of the process, a single assignment was obtained for each sequence with a 97.5% identity, 90 bp minimum alignment length, and from 16 to 16,382 supporting reads (number of reads clustered together to generate the consensus sequence).

### 2.6. Data Analyses

The observed number of species was defined as (S). S for mosquitoes was calculated for each site and S of host-blood-meal source was also defined for each mosquito species. The ‘human blood index’ (HBI) refers to the proportion of mosquito blood meals originating from humans and serves as a crucial factor in determining pathogen transmission risk. To investigate the degree of anthropophily among mosquitoes, the human blood index (HBI) was therefore calculated for each species as follows: Human blood Index = Number of mosquitoes that have fed on humans divided by total number of mosquitoes whose blood meals have been identified [32,33].

Agreement between the results of Sanger sequencing and MinION sequencing was measured using Cohen’s Kappa measure of test association [34] in STATA software. When the Kappa value is less than 0.20, 0.21–0.40, 0.41–0.60, 0.61–0.80, and over 0.81, their agreements are rated as no agreement/slight, fair, moderate, substantial, and almost perfect, respectively.

Nanopore MinION sequencing analysis identified single and multiple blood-meal sources. We combined Nanopore and Sanger sequencing to obtain confident vertebrate species detection from blood-fed females in French Guiana.

At the mosquito community level, to analyze possible blood-source selection by the mosquito species community, a network analysis was performed. The Jenks natural breaks algorithm was employed to divide the dataset into homogenous classes of species based on their abundance [35]. The class with the lowest number of individuals was excluded from the network community analysis. Then, an interaction-weighted matrix was constructed, with mosquito species and vertebrates identified through blood source identification. The values in this matrix corresponded to the number of identified blood meals from each vertebrate species for each mosquito species. The open-source R package BipartiteR was utilized to produce a bipartite graph of the samples based on linkage density. This analysis identified interaction modules that are useful for indicating aggregated patterns of behavior [36].

## 3. Results

### 3.1. Blood-Fed-Female Collection and Identification

**Figure 2:**
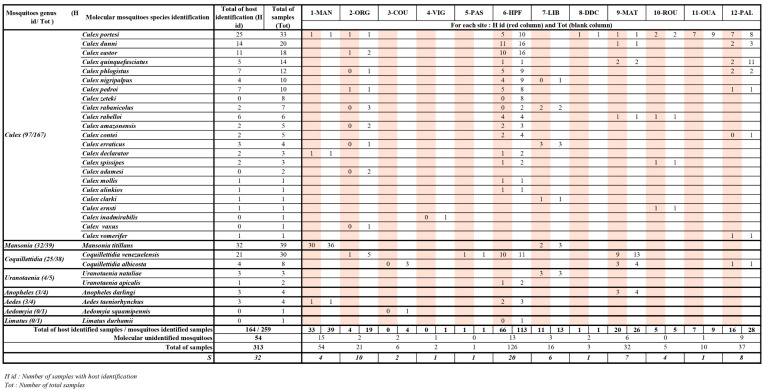
Number of samples per mosquito species and per site with molecular identification of the blood-fed females and their associated blood meal(s). For each genus, mosquito species are organized from highest to lowest sample numbers. Species richness is indicated for each locality and habitat. Sites are organized from highest to lowest species richness by habitat. The collected 12 French Guiana localities are 6-HPF (Hameau Préfontaine), 12-PAL (Trois Palétuviers), 7-LIB (Pointe Liberté), 10-ROU (Roura), 11-OUA (Ouanary), 8-DDC (Dégrad des Cannes), 2-ORG (Organabo), 3-COU (Counamama), 4-VIG (Viguié), 5-PAS (Passoura), 9-MAT (Matoury), and 1-MAN (Mana). The total numbers of samples and those with host identification are also indicated.

Among the 32868 mosquitoes trapped over a 5-year period, a total of 313 of blood-fed females were collected from 12 sites. Most specimens belong to the *Culex* (Cx.) genus (59%), followed by seven other genera: *Mansonia* (Ma.) (17%), *Coquillettidia* (Cq.) (14%), *Uranotaenia* (Ur.) (5%), *Aedes* (Ae.) (2%), *Anopheles* (An.) (1%), *Limatus* (Li.) (1%), and *Aedeomyia* (Ad.) (1%). Molecular barcoding of blood-fed females based on Sanger sequencing of the COI fragments allowed the identification to species level of 259 specimens (84% of the collection). The five most abundant species were *Ma. titillans* with 12% (32/259), *Cx. portesi* with 10% (25/259), *Cq. venezuelensis* with 8% (21/259), *Cx. dunni* with 5% (14/259), and *Cx. eastor* with 4% (11/259) (Figure 2).

### 3.2. Blood-Meal-Source Species Identification

Mosquito specimens, identified at the species level, underwent analyses to identify the species of host-blood meals. A total of 47% (123/259) of blood-meal sources were identified when 136/259 of samples failed (Figure S1). Using the same PCR product, a random subset of 193 samples (103 undetermined and 90 previously identified) were also analyzed with MinION nanopore sequencing. A total of 82 samples already identified using the Sanger sequencing method were also identified with the MinION nanopore sequencing method, and 62 samples that were not identified with the Sanger sequencing method persisted unidentified with the MinION nanopore sequencing method. The remaining samples were identified only with the Sanger method (8/193) or others only with the MinION nanopore sequencing method (41/193). The agreement between the two methods was defined as almost perfect by the Kappa statistic (k = 1) (Figure S1). Finally, 63% (164/259) of all female mosquitoes, representing 26 species across 6 genera, had their blood-meal source identified at the species level. A detailed workflow describing the step-by-step results of molecular identifications of female mosquitoes and vertebrate origins of blood meals is presented as supplementary data in Figure S1.

The distribution by habitat of all blood-fed females identified to species level is indicated in Figure 2. The site with the highest mosquito species richness observed was in the site of Macouria (6-HPF), which also had the most important sampling effort. *Culex portesi* was the only species found across all type of habitat and in majority of the sites (8/12). Figure 2 indicates the numbers of samples with host identification by site and mosquito species.

### 3.3. Multiple Blood-Meal-Source Identification

**Figure 3:**
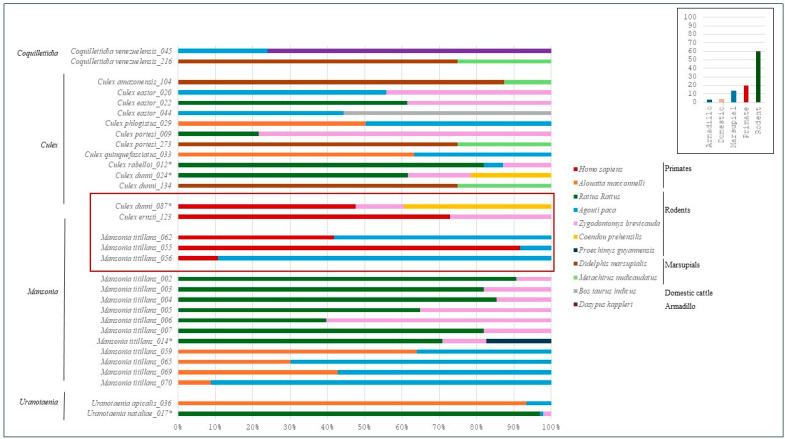
Multiple-blood-meal structure inferred from the number of reads obtained for each host species. The red box highlights samples containing human blood meals. A star “*” next to sample indicates a sample with three blood-meal sources identified. Cumulative number of reads by type of host are visible in the box in the top right corner.

The MinION nanopore sequencing method was used for detection and identification of multiple blood meals. A total of 31 samples, corresponding to 12 species of mosquitoes, were found with multiple blood meals (Figure 3). The majority, 84% (26/31), consisted of two different blood meals, but five blood meals were found with three host species. Only mammals were found in the multiple composition of blood meals, including humans, non-human primates, rodents, marsupials, domestic cattle, and armadillos. Human blood meals were found mixed with other mammals in five samples from three different species: three *Ma. titillans*, one *Cx. dunni*, and one *Cx. ernsti*. All samples were mixed with rodents. An analysis of the number of reads by host type shows mostly rodent reads (60% of the total number of reads) (Figure 3).

### 3.4. Host-Blood-Meal Pattern Analysis

A total of 200 associations, including 164 samples with both mosquito species identification and blood-meal-source identification and the 36 mosquito–host associations adding value from multiple blood-meal sources, were used for the analysis (Figure S1).

**Figure 4:**
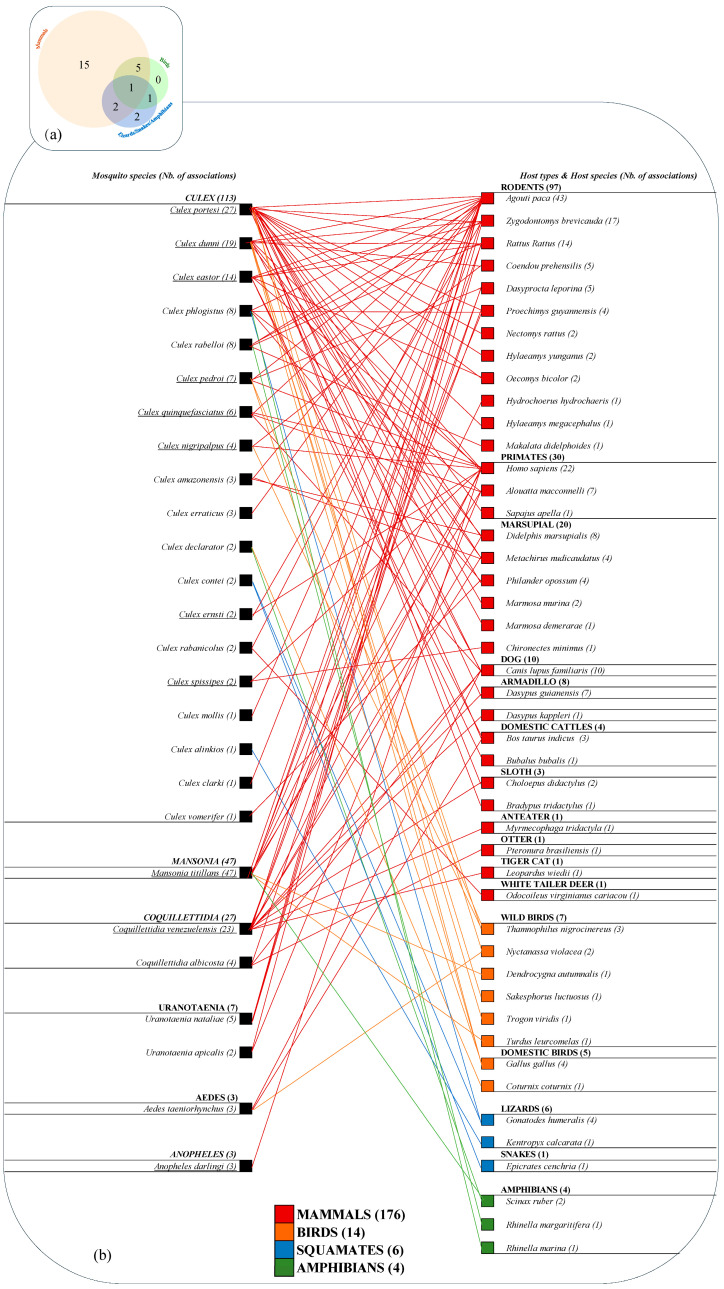
Distribution of mosquito species according to their blood-meal composition. (**a**) Venn diagram of the distribution of the number of mosquitoes species by class of host; (**b**) Composite image illustrating mosquito and host associations from the study. The number of associations is indicated within brackets. Species are ordered according to their number of associations, from the highest to the lowest. Red links correspond to mammals, orange links to birds, blue links to lizards and snakes, and green links to amphibians. Mosquito species found with human blood are underlined.

All species of mosquitoes fed on mammals (Figure 4a,b), except three species. From this dataset, exclusive mammal feeding was observed for 15 species (Figure 4a). Cold-blood feeding (amphibians/lizards/snakes) was detected for six species and strict cold-blood feeding was only noted in two species: *Cx. contei* and *Cx. alinkios*. All bird feeding was not exclusive and mosquitoes also fed either on mammals or on mammals and amphibians/lizards/snakes (Figure 4a). *Culex portesi*, *Cx. dunni*, *Cx. pedroi*, *Cx. nigripalpus*, *Cx. declarator*, *Ma. titillans*, and *Ae. taeniorhynchus* were found to have a bird-blood meal (Figure 4a,b). Two Culex species (*Cx. rabelloi* and *Cx. phlogistus*) fed on mammals and on amphibians/lizards/snakes. *Culex declarator* fed on birds and amphibians. Finally, no species were found to have fed on mammals, birds, amphibians, and squamates.

The host-type association with each mosquito species (Figure 4b) reveals that most mosquito species (22/28) fed mainly on rodents, with 50% of associations (97/195). Humans were the second most highly detected host species in all samples with 12% (23/195) of associations, followed by marsupials with 8% (16/195), and dogs with 5% (10/195).

**Figure 5:**
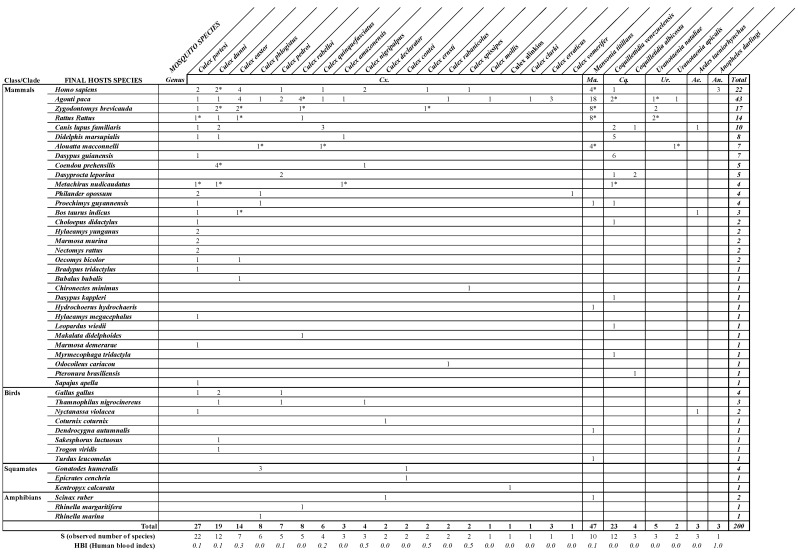
Distribution of samples for each mosquito species according to the source species of host-blood meals, organized by class and by highest to lowest number. Observed number of species and human blood index (HBI) are provided at the bottom, for each mosquito species. A star “*” is present next to samples that include multiple blood meals.

More specifically (Figure 5), the predominant mammalian species were the lowland paca (*Agouti paca*) (22%), followed by humans (11%), the short-tailed cane mouse (*Zygodontomys brevicauda*) (9%), black rats (*Rattus rattus*) (7%), and dogs (*Canis lupus familiaris*) (5%). The ‘human blood index’ (HBI) was calculated for each species. Humans were found to be a source of blood meal for 11 species of mosquitoes, with an HBI ranging from 0.04 to 1. *Culex eastor*, *Ma. titillans*, and *An. darlingi* were the primary species found with human blood. *Culex portesi* showed the highest diversity in hosts used for feeding as it fed on 22 different species. Bird species identified included mainly chickens (*Gallus gallus*) and blackish-grey antshrikes (*Thamnophilus nigrocinereus*), representing 29 and 21% of the total bird species, respectively. Amphibians were represented by the red-snouted treefrog (*Scinax ruber*) (50%) and species of South American toads (*Rhinella* genus) (50%). Details on the range of host species by mosquito species are found in Figure 5.

### 3.5. Network Analysis

**Figure 6:**
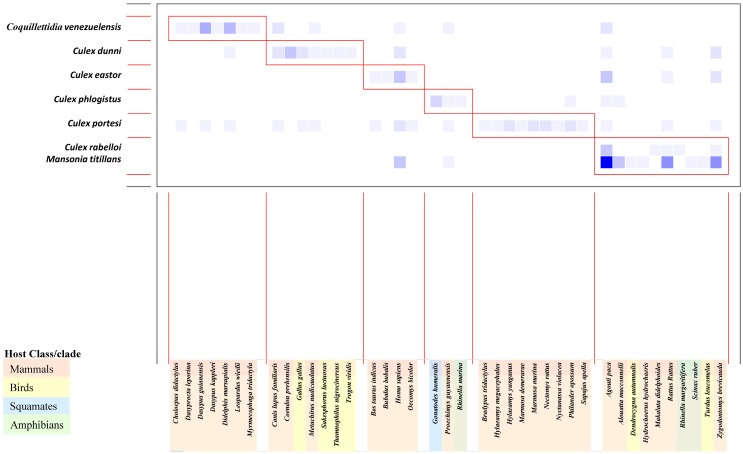
Interaction matrix highlighting modular relationships between vertebrate blood-meal sources and adult female mosquitoes that fed upon them. Boxes delineate modules, with darker squares indicating a greater number of detected interactions, gradually transitioning to lighter colors to represent fewer interactions detected. Host classes/clades are indicated by color codes as shown in the figure.

Analysis of the bipartite mosquito–vertebrate network comprised 72 different host–mosquito interactions (Figure 6) and detected six modules of mosquito species interacting more frequently with specific subsets of vertebrates. Five modules were associated with a single mosquito species, meaning they showed a very different behavior compared to other species. *Coquillettidia venezuelensis*, *Culex eastor*, and *Culex portesi* were only associated with mammals, *Culex dunni* was associated with mammals and birds, while *Cx. phlogistus* was associated with mammals and amphibians/squamates. The remaining module gathered two species of mosquitoes (*Cx. rabelloi* and *Ma. titillans*) and was associated with mammals, birds, and amphibians. Humans were grouped in the same module as domestic cattle and rodents.

## 4. Discussion

### 4.1. High Mammal-Blood-Meal Detection

The host feeding patterns of mosquitoes vary depending on the species [37]. Some mosquito species are relatively generalist, while others show some degrees of specialization, focusing on a particular subset or individual members of the available host community. The present study indicated that most of the mosquito species analyzed shared one or more host species. Although we detected 46 host species, there was a distinctly higher frequency of mammals and especially lowland pacas, humans, short-tailed cane mice, black rats, and dogs. More than 62% of the mosquito species shared a similar host species: the lowland paca rodent. Host selection of mosquitoes towards this mammal is certainly due to its relative abundance in a wide array of sampled ecosystems such as different kinds of tropical forests, mangroves, and riverine vegetation [38] and its relatively tolerance to habitat fragmentation and land use change in the tropical forests [39,40]. Their large body size makes them an accessible target for mosquito biting and potentially ideal prey, especially if coupled with low defensive behavior. Their nocturnal activity out of burrows is more likely to attract mosquitoes, which were primarily captured at night in this study, drawn by physical and chemical cues released through breath and skin activity. While most of the mammalian hosts detected were nocturnal, diurnal species were also found in our sampling, including humans, dogs, and monkeys. These interactions are likely due to contacts occurring during common activity periods of the day, such as crepuscular periods or at dawn.

### 4.2. Few Bird Feeders and No Strict Ornithophilic Species

Only seven mosquito species were found to have bird blood in their blood meal: namely, *Ma. titillans*, *Ae. taeniorhynchus*, and five species of *Culex* genus: *Cx. portesi*, *Cx. dunni*, *Cx. pedroi*, *Cx. Nigripalpus*, and *Cx. declarator*. *Culex* species are generally thought to feed on birds [41,42]. In reality, the feeding patterns of only relatively few species of this large genus are known [37]. Nevertheless, no bird meal was found in several mosquito species already identified in the literature as feeding on birds. Identification was performed using a short marker, with highly conserved binding sites, located in the mitochondrial 12S ribosomal RNA, designed for the metabarcoding of vertebrates and validated for accurate taxonomic assignments for Amazonian mammals [28]. The accuracy of a marker often depends on a reliable molecular reference database [28]. For example, the NCBI’s 12S gene reference database sequences of mammals contain three times more sequences than those of birds, despite there being 200 known mammal species compared to 698 bird species in French Guiana. This potential lack of bird reference sequences could introduce biases in the identification process, making it difficult to identify some specimens at the species level. The use of additional genetic markers could further enhance the resolution of detecting various host-blood-meal sources, as a multi-faceted approach is always preferable for more accurate identification. Additionally, mosquito samplings were conducted at ground level using CDC light traps or manual aspiration in resting sites, which may also lead to an underrepresentation of female mosquitoes that may have fed on birds in the canopy, for example. Moreover, some mosquito species can modify their feeding behavior depending on host availability or even the season. For example, *Cx. nigripalpus* shows a feeding shift from ornithophilic to opportunistic in the USA, with a proportional increase in feeding on mammals occurring in summer and then shifting back to avian hosts, which dominate the feeding pattern during winter and spring [7,43,44]. Consequently, *Cx. nigripalpus* is likely an early-summer-season amplification vector, circulating the virus among avian hosts at the beginning of the season, and a late-season bridge vector, spreading infections from avian to mammalian hosts in the epidemic cycle [44]. The same applies to *Cx. erraticus*, which primarily feeds on avian hosts in the spring and summer months, then switches to feeding primarily on mammalian hosts in the summer and fall [45,46]. Therefore, it would be interesting to revisit our various capture sites to study these probable temporal variations in trophic preference. Nonetheless, we successfully detected four classes of vertebrates across all our samples, and the presence of these vertebrates in these locations was coherent with their known ecology and distribution.

### 4.3. Generalist Feeder as a Potential Bridge Vector

The two most abundant mosquito species, *Ma. titillans* and *Cx. portesi*, appear to be true generalists and opportunistic in their blood-feeding behavior. *Mansonia titillans* is widely distributed in America and is known to preferentially bite humans [47,48], but also feeds on domestic and wild vertebrate hosts such as pigs or horses [49]. In French Guiana, this species is known for its aggressive behavior towards humans [50]. In our study, *Ma. titillans* was found to feed on humans, but its preferred feeding hosts were rodents, in decreasing order: lowland pacas (*Agouti paca*), short-tailed cane mice (*Zygodontomys brevicauda*), and black rats (*Rattus rattus*). *Zygodontomys* is considered a reservoir during the enzootic transmission cycle of the Venezuelan equine encephalitis virus (VEEV, Togaviridae: Alphavirus) [51] and *Ma. titillans* have already been found to be naturally infected with this virus [52]. This mosquito is also suspected to be a bridge vector of St. Louis encephalitis virus (SLEV, Flaviviridae: Orthoflavivirus), carrying the virus from sylvatic/rural to peridomestic environments where it could infect humans [52,53,54]. The Bunyamwera serogroup, one of the most significant serogroups in the Orthobunyavirus genus [55], was discovered in a pool of *Ma. titillans* in Argentina [54]. Furthermore, we frequently observed in this species the occurrence of multiple blood meals within a single gonotrophic cycle, with more than half of the samples tested via NGS indicating a mixed meal. Three dual-blood feedings involved a combination of rodents and humans. Despite a strong preference for rodents, *Ma. titillans* have also been found to feed on birds, amphibians, and non-human primates. The species is also aggregated in a module with *Cx. rabelloi* species, underlying an unspecific behavior. Unlike our findings, other studies indicate proportions of blood meals from birds and humans to be higher than those from rodents in Rio de Janeiro [56]. Drawing preferential conclusions is always challenging since blood feeding sources vary among localities and are likely influenced by host availability. Nevertheless, taken together, these results suggest that the species exhibits highly opportunistic feeding behavior. The use of NGS sequencing technologies in blood-meal analysis has been heralded as a tool of the future for detecting new or unexpected hosts, enhancing our understanding of mosquito feeding habits. However, we are fully aware that PCR-based methods using NGS are highly sensitive to laboratory contamination and must be interpreted with caution. Adequate negative controls are essential to determine detection limits and minimize laboratory or sequencing artifacts.

*Culex portesi* is a species distributed only in Amazonia and frequently found along the coastal plain of French Guiana in all habitats [23]. This species is highly opportunistic, biting mammals and birds. Several viruses (e.g., Bimiti, VEEV, Mucambo, Cabassou, Caraparu, Itaqui, Tonate, Marituba, and Maguari) were isolated from this species in Brazil, French Guiana, Suriname, and Trinidad [57]. In French Guiana, the viruses of the VEE complex are mainly transmitted by *Cx. portesi* and are thought to have rodent and marsupial vertebrates as reservoir hosts [49]. In our study, rodents and marsupials were highly detected as the predominant blood source. *Culex portesi* has a long gonotrophic cycle in natural conditions, favorable for the multiplication and transmission of arboviruses. This extended cycle allows the virus to replicate within the mosquito for an extended period, increasing the likelihood of transmission to other hosts during subsequent blood feedings. This mosquito species is also able to retain mature eggs in the absence of suitable oviposition sites and to survive for long periods in the absence of a blood-meal source. As already described by Dégallier (1982) [49], this study reinforces the idea that this species should be able to play a significant role as a vector. Indeed, the species behavior displays many key characteristics: it is an opportunistic feeder, multiple blood meals are taken during a single gonotrophic cycle, it has an impressive range of hosts including humans, and it has diverse habitats along with a capacity to transmit arboviruses. *Culex portesi* is known to play a role in sylvatic viral epizootics and therefore deserves surveillance. Indeed, generalist species are less likely to transmit a specific pathogen to a single host than specialist feeders but are more likely to act as bridge vectors for zoonotic infections by transmitting a pathogen from a reservoir host to other susceptible hosts, including humans [58]. In South America, the genus *Anopheles* is the most widely studied in terms of publications, number of individuals, and geographic scope [2], as several species of the genus are implicated in human malaria parasite transmission. Although most studies have been conducted near human habitations, it seems that *Anopheles* express a clear preference for mammals, mainly large mammals (humans, cattle). In forests, where the diversity of potential hosts is high, *Anopheles* become more generalist and females can bite several species of mammals [59,60,61]. In French Guiana, *Anopheles darlingi* is the main vector of human malaria [62,63]. This anthropophilic behavior was confirmed with *An. darlingi* samples of the collection that had the highest human blood index.

### 4.4. Abundant Culex Species in Collection, Nearly Half Detected with Human Blood

We obtained 88% of samples belonging to the *Culex* genus in all collected habitat types with 8 species (out of 19) with human blood-meal origin. The feeding pattern of only a few species of this large genus are known, particularly in South America as recently highlighted by Melgarejo-Colmenares and collaborators [2]. The synthesis of data obtained until 2020 from this publication allowed the identification of the trophic preference of only seven species of *Culex*, whereas, for example, in French Guiana, there are over 100 species [23]. The trophic preferences of these mosquitoes are poorly understood because of the difficulty associated with the morphological identification of *Culex* females, which is now made possible through molecular identification. The most common species in French Guiana, and widely distributed in the world, is *Cx. quinquefasciatus*. This species is strongly associated with human environments [64]. *Culex quinquefasciatus* was found to have fed on humans, dogs, lowland paca, and a lizard. Other sources such as birds, horses, and cattle have also been found in other studies [65,66,67,68]. The preferred blood-meal sources of this species appear to vary in different regions, possibly due to differences in host abundance and availability. Numerous arboviruses have been identified in this species, including WNV [69] and SLEV [70]. Detection of *Cx. quinquefasciatus* across French Guiana occurred primarily in urban areas, although it is not uncommon to find them in peri-urban and rural areas as well. Their feeding behavior suggests the potential to serve as a bridge vector for transmitting pathogens across multiple taxa. Human blood has also been detected in *Cx. spissipes*. This species is already recognized as a potential vector for various viruses of the Peribunyaviridae family (such as Bimiti, Caraparu, Oriboca, and Itaqui viruses) and the Togaviridae family (including VEEV and Mucambo viruses) [52,71,72]. *Culex pedroi* serves as a vector for Eastern equine encephalitis virus in Brazil and Trinidad [73], as well as for VEEV and other viruses in Panama [52,74,75]. Maybe due to the very small horse industry in French Guiana, no horses have been detected thus far as sources for *Cx. pedroi*, but several mammals have, including humans, which is similar to the findings in Trinidad. The first detection of human blood meals was identified for three mosquito species of French Guiana: *Culex dunni*, *Cx. eastor*, and *Cx. ernsti*. *Culex dunni* also fed on birds. Indeed, the Pacora virus (PAC: Bunyaviridae: Bunyavirus-like) was detected in *Cx. dunni* in Panama, and from two sylvatic birds in Brazil, probably suggesting a sylvatic bird–*Culex* cycle. This *Culex* species is also capable of intermittent meals, up to three different meals within the same gonotrophic cycle, as observed in this study.

### 4.5. Multiple Blood Meals

Studies have shown that mixed or multiple blood meals can account for up to 10% of mosquitoes in the field [61,76]. In our collection, 17% of sample collections contained multiple blood meals from 11 mosquito species. These data are epidemiologically significant for the transmission of human pathogens, as mosquito vectors may feed on an animal or a person, and subsequently, on other humans. A well-established example is that several anopheline species take multiple blood meals during each gonotrophic cycle [77,78,79,80], a behavior that can affect vectorial capacity. Virus-infected mosquitoes that take an additional non-infectious blood meal are more likely to disseminate and transmit the virus than those feeding only once, as blood meals may cause microperforations in the basal lamina surrounding the midgut, aiding viral escape [81]. Moreover, arboviral transmission efficiency can be influenced by the blood source; for instance, USUV infection rates in *Culex pipiens* were significantly higher with infectious human blood than with infectious chicken blood [82]. Incorporating multiple blood-meal detection methods in studies can help more accurately estimate the frequency of feeding on multiple vertebrate species, which is a key parameter in understanding vector behavior and a vector’s capacity to spread diseases.

## 5. Conclusions

In this study, we expanded the list of blood-meal sources of neotropical mosquito species, aiming to improve the knowledge of the relationships between the highly diverse communities of vertebrates and mosquitoes present in French Guiana. NGS-based approaches to blood-meal analysis should be encouraged, as they help investigate multiple blood-meal habits. Given the limited information available on host preferences for most mosquito species in South America, understanding the patterns of host use among Culicidae can shed light on various aspects of mosquito ecology and behavior that are not well understood for some species, and assess behaviors that can bridge arboviral transmission across different vertebrate groups. Furthermore, continuous monitoring of mosquito host use is essential to detect any shifts in behavior or host preferences that could indicate a higher risk of disease transmission to humans.

## Data Availability

The datasets of the current study are available in Supplementary Table S2 and all other data are available on request from the corresponding author.

## Supplementary Materials

The following are available online at https://www.mdpi.com/article/10.3390/insects15090718/s1, Table S1: Field collection data by site. Figure S1. Workflow of the methodological approach and results obtained for female molecular identifications and blood-meal analyses used in the study. Table S2: Detailed overview of blood-fed female mosquito samples: localization, mosquitoes, and blood-meal results by Sanger and MinION sequencing with final identification.
