# Supplementary material for: Mosquito Feeding Habits in Coastal French Guiana: Mammals in the Crosshairs?
Source: Insects. 2024 Sep 19;15(9):718. doi: 10.3390/insects15090718 (PMC11432726; doi:10.3390/insects15090718)
Supplement: Supplementary file 1 [file insects-15-00718-s001.zip › FigureS1.pdf]

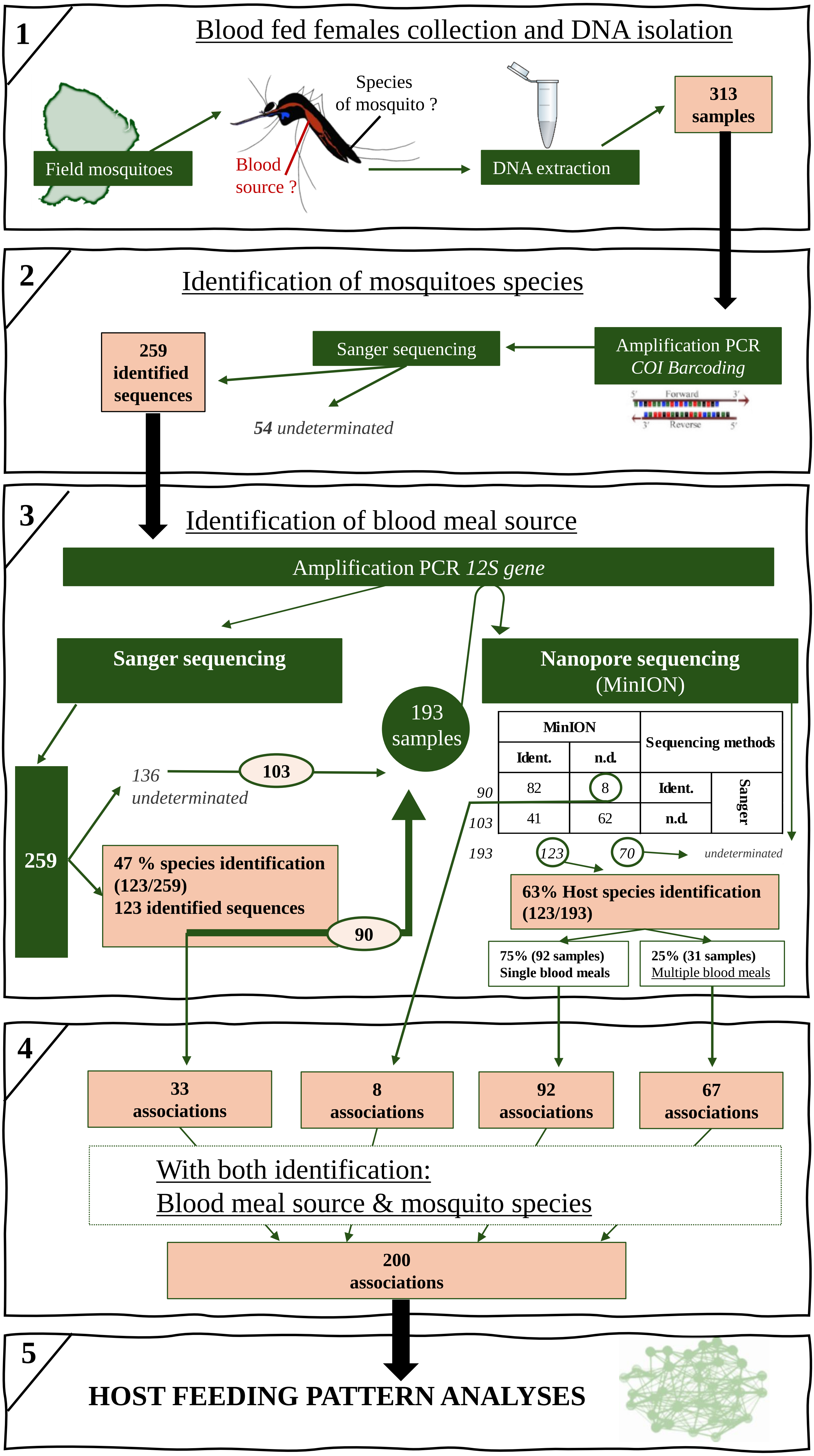
1
Blood fed females collection and DNA isolation
Field mosquitoes
Species of mosquito ?
Blood source ?
DNA extraction
313 samples
2
Identification of mosquitoes species
Amplification PCR COI Barcoding
Forward
Reverse
Sanger sequencing
259 identified sequences
54 undeterminated
3
Identification of blood meal source
Amplification PCR 12S gene
Sanger sequencing
Nanopore sequencing (MinION)
193 samples
259
136 undeterminated
103
47 % species identification (123/259) 123 identified sequences
90
MinION
Sequencing methods
Ident.
n.d.
Sanger
90
82
8
Ident.
103
41
62
n.d.
193
123
70
undeterminated
63% Host species identification (123/193)
75% (92 samples) Single blood meals
25% (31 samples) Multiple blood meals
4
33 associations
8 associations
92 associations
67 associations
With both identification:
Blood meal source & mosquito species
200 associations
5
HOST FEEDING PATTERN ANALYSES
