# Supplementary material for: Mosquito Feeding Habits in Coastal French Guiana: Mammals in the Crosshairs?
Source: Insects. 2024 Sep 19;15(9):718. doi: 10.3390/insects15090718 (PMC11432726; doi:10.3390/insects15090718)
Supplement: Supplementary file 1 [file insects-15-00718-s001.zip › TableS1.pdf]

Table S1: Field collection data by site. The table summarizes the number of blood-fed female mosquitoes collected at each site, including collection dates, times, trap types, and total mosquitoes per site (cumulative for multiple sessions).

Sites	Blood-fed females nb.	Month and year of collection	Traps and Time of collection of blood fed females	Total mosquitoes collection
**1-MAN**	54	Oct. 2018 and Mar. 2019	CDC-light with and without dry ice (6:00 PM to 8:00 AM)	4601
**2-ORG**	21	Aug. 2022	CDC-UV-light (5:00 PM to 8:00 AM)	966
**3-COU**	6	Aug. 2022	CDC-UV-light (5:00 PM to 8:00 AM)	1642
**4-VIG**	2	Aug. 2022	CDC-UV-light (5:00 PM to 8:00 AM)	89
**5-PAS**	1	Aug. 2022	CDC-UV-light (5:00 PM to 8:00 AM)	329
**6-HPF**	126	Dec. 2018; Mar. 2019 and Jan. 2020	CDC-light with and without dry ice (6:00 PM to 8:00 AM)	13416
**7-LIB**	16	Nov. 2018 and Mar. 2019	CDC-light with and without dry ice (4:00 PM to 8:00 AM)	4046
**8-DDC**	3	Avr. 2019	CDC-light with and without dry ice (5:00 PM to 8:00 AM)	2678
**9-MAT**	32	Jul. 2023	Octenol Mosquito Magnet (5:00 PM to 7:00 AM)	185
**10-ROU**	5	Oct. 2018	CDC-light (6:00 PM to 9:00 AM) and Aspiration (8:00 AM à 9:00 AM)	1193
**11-OUA**	10	Nov. 2022	CDC-UV-light (5:00 PM to 8:00 AM)	1200
**12-PAL**	37	Aug. 2018	CDC-light (5:00 PM to 7:00 AM)	2523
	313			*32868*
