# Supplementary material for: Mosquito Feeding Habits in Coastal French Guiana: Mammals in the Crosshairs?
Source: Insects. 2024 Sep 19;15(9):718. doi: 10.3390/insects15090718 (PMC11432726; doi:10.3390/insects15090718)
Supplement: Supplementary file 1 [file insects-15-00718-s001.zip › TableS2.pdf]

Sample localization				Mosquito species identification			Host blood meal identification									
**Samples number**	**Localization**	**Lattitude_Y**	**Longitude_X**	**Mosquito taxonomic genus**	**Molecular identification of mosquitoes (COI, Sanger sequencing)**	**Accession number**	**Blood meal identification (using Sanger sequencing)**	**Accession number**	**Sample test with MINION**	**Blood meal identification (using Nanopore MinION sequencing)**	**Final supporting reads**	**Multiple blood meal**	**FINAL BLOOD MEAL IDENTIFICATION**	**Classe**	**Ordre**	**Host Types**
**Biome_G_001**	MAN	5.632552547405859	-53.66091898281266	*Mansonia*	***Ma. (Man.) titillans***	MT999303.1	*n.d.*		oui	*Zygodontomys brevicauda*	2216	non	***Zygodontomys brevicauda***	Mammals	Rodentia	Rodent
**Biome_G_002**	MAN	5.632552547405859	-53.66091898281266	*Mansonia*	***Ma. (Man.) titillans***	MT999303.1	*n.d.*		oui	*Rattus Rattus*	8340	oui	***Rattus Rattus***	Mammals	Rodentia	Rodent
**Biome_G_002**	MAN	5.632552547405859	-53.66091898281266	*Mansonia*	***Ma. (Man.) titillans***	MT999303.1	*n.d.*		/	*Zygodontomys brevicauda*	860	oui-m2	***Zygodontomys brevicauda***	Mammals	Rodentia	Rodent
**Biome_G_003**	MAN	5.632552547405859	-53.66091898281266	*Mansonia*	***Ma. (Man.) titillans***	MT999303.1	*n.d.*		oui	*Rattus Rattus*	2470	oui	***Rattus Rattus***	Mammals	Rodentia	Rodent
**Biome_G_003**	MAN	5.632552547405859	-53.66091898281266	*Mansonia*	***Ma. (Man.) titillans***	MT999303.1	*n.d.*		/	*Zygodontomys brevicauda*	542	oui-m2	***Zygodontomys brevicauda***	Mammals	Rodentia	Rodent
**Biome_G_004**	MAN	5.632552547405859	-53.66091898281266	*Mansonia*	***Ma. (Man.) titillans***	MT999303.1	*n.d.*		oui	*Rattus Rattus*	5980	oui	***Rattus Rattus***	Mammals	Rodentia	Rodent
**Biome_G_004**	MAN	5.632552547405859	-53.66091898281266	*Mansonia*	***Ma. (Man.) titillans***	MT999303.1	*n.d.*		/	*Zygodontomys brevicauda*	1026	oui-m2	***Zygodontomys brevicauda***	Mammals	Rodentia	Rodent
**Biome_G_005**	MAN	5.632552547405859	-53.66091898281266	*Mansonia*	***Ma. (Man.) titillans***	MT999303.1	*n.d.*		oui	*Rattus Rattus*	2690	oui	***Rattus Rattus***	Mammals	Rodentia	Rodent
**Biome_G_005**	MAN	5.632552547405859	-53.66091898281266	*Mansonia*	***Ma. (Man.) titillans***	MT999303.1	*n.d.*		/	*Zygodontomys brevicauda*	1464	oui-m2	***Zygodontomys brevicauda***	Mammals	Rodentia	Rodent
**Biome_G_006**	MAN	5.632552547405859	-53.66091898281266	*Mansonia*	***Ma. (Man.) titillans***	MT999303.1	*n.d.*		oui	*Zygodontomys brevicauda*	2500	oui	***Zygodontomys brevicauda***	Mammals	Rodentia	Rodent
**Biome_G_006**	MAN	5.632552547405859	-53.66091898281266	*Mansonia*	***Ma. (Man.) titillans***	MT999303.1	*n.d.*		/	*Rattus Rattus*	1650	oui-m2	***Rattus Rattus***	Mammals	Rodentia	Rodent
**Biome_G_007**	MAN	5.632552547405859	-53.66091898281266	*Mansonia*	***Ma. (Man.) titillans***	MT999303.1	*n.d.*		oui	*Rattus rattus*	410	oui	***Rattus Rattus***	Mammals	Rodentia	Rodent
**Biome_G_007**	MAN	5.632552547405859	-53.66091898281266	*Mansonia*	***Ma. (Man.) titillans***	MT999303.1	*n.d.*		/	*Zygodontomys brevicauda*	90	oui-m2	***Zygodontomys brevicauda***	Mammals	Rodentia	Rodent
**Biome_G_008**	MAN	5.632552547405859	-53.66091898281266	*Mansonia*	***Ma. (Man.) titillans***	MT999303.1	*n.d.*		oui	*Scinax ruber*	168	non	***Scinax ruber***	Amphibians	Anura	Amphibian
**Biome_G_009**	MAN	5.632552547405859	-53.66091898281266	*Culex*	***Cx. (Mel.) portesi***	FGMOS2416-20	*n.d.*		oui	*Zygodontomys brevicauda*	1164	oui	***Zygodontomys brevicauda***	Mammals	Rodentia	Rodent
**Biome_G_009**	MAN	5.632552547405859	-53.66091898281266	*Culex*	***Cx. (Mel.) portesi***	FGMOS2416-20	*n.d.*		/	*Rattus rattus*	320	oui-m2	***Rattus Rattus***	Mammals	Rodentia	Rodent
**Biome_G_010**	MAN	5.632552547405859	-53.66091898281266	*Culex*	***Cx. (Cux.) declarator***	FGMOS2281-20	*Scinax ruber*	AY549365.1	oui	*Scinax ruber*	5738	non	***Scinax ruber***	Amphibians	Anura	Amphibian
**Biome_G_011**	MAN	5.632552547405859	-53.66091898281266	*Aedes*	***Ae. (Och.) taeniorhynchus***	MW339699.1	*Nyctanassa violacea*	MW524627.1	oui	*Nyctanassa violacea*	21500	non	***Nyctanassa violacea***	Birds	Pelecaniformes	Wild bird
**Biome_G_012**	ROU	4.741465576104164	-52.32568961154607	*Culex*	***Cx. (Mel.) rabelloi***	FGMOS2230-20	*n.d.*		oui	*Rattus rattus*	3550	oui	***Rattus Rattus***	Mammals	Rodentia	Rodent
**Biome_G_012**	ROU	4.741465576104164	-52.32568961154607	*Culex*	***Cx. (Mel.) rabelloi***	FGMOS2230-20	*n.d.*		/	*Zygodontomys brevicauda*	564	oui-m2	***Zygodontomys brevicauda***	Mammals	Rodentia	Rodent
**Biome_G_012**	ROU	4.741465576104164	-52.32568961154607	*Culex*	***Cx. (Mel.) rabelloi***	FGMOS2230-20	*n.d.*		/	*Agouti paca*	220	oui-m3	***Agouti paca***	Mammals	Rodentia	Rodent
**Biome_G_013**	LIB	4.915387042086516	-52.3613138909788	*Culex*	***Cx. (Cux.) nigripalpus***	FGMOS2667-20	*n.d.*		non	Pas de minion	Pas de minion	/	***n.d.***	*n.d.*	*n.d.*	*n.d.*
**Biome_G_014**	MAN	5.632552547405859	-53.66091898281266	*Mansonia*	***Ma. (Man.) titillans***	MT999303.1	*n.d.*		oui	*Proechimys guyannensis*	1578	oui	***Proechimys guyannensis***	Mammals	Rodentia	Rodent
**Biome_G_014**	MAN	5.632552547405859	-53.66091898281266	*Mansonia*	***Ma. (Man.) titillans***	MT999303.1	*n.d.*		/	*Rattus rattus*	6410	oui-m2	***Rattus Rattus***	Mammals	Rodentia	Rodent
**Biome_G_014**	MAN	5.632552547405859	-53.66091898281266	*Mansonia*	***Ma. (Man.) titillans***	MT999303.1	*n.d.*		/	*Zygodontomys brevicauda*	1066	oui-m3	***Zygodontomys brevicauda***	Mammals	Rodentia	Rodent
**Biome_G_015**	LIB	4.915387042086516	-52.3613138909788	*Mansonia*	***Ma. (Man.) titillans***	MT999303.1	*Turdus leucomelas*	EU154524.1	oui	*Turdus leucomelas*	39050	non	***Turdus leucomelas***	Birds	Passeriformes	Wild bird
**Biome_G_016**	LIB	4.915387042086516	-52.3613138909788	*Uranotaenia*	***Ur. (Ura.) nataliae***	FGMOS2905-22	*Zygodontomys brevicauda*	KX381438.1	oui	*Zygodontomys brevicauda*	1397	non	***Zygodontomys brevicauda***	Mammals	Rodentia	Rodent
**Biome_G_017**	LIB	4.915387042086516	-52.3613138909788	*Uranotaenia*	***Ur. (Ura.) nataliae***	FGMOS2905-22	*Zygodontomys brevicauda*	KX381438.1	oui	*Zygodontomys brevicauda*	694	oui	***Zygodontomys brevicauda***	Mammals	Rodentia	Rodent
**Biome_G_017**	LIB	4.915387042086516	-52.3613138909788	*Uranotaenia*	***Ur. (Ura.) nataliae***	FGMOS2905-22	*n.d.*		/	*Rattus Rattus*	30720	oui-m2	***Rattus Rattus***	Mammals	Rodentia	Rodent
**Biome_G_017**	LIB	4.915387042086516	-52.3613138909788	*Uranotaenia*	***Ur. (Ura.) nataliae***	FGMOS2905-22	*n.d.*		/	*Agouti paca*	254	oui-m3	***Agouti paca***	Mammals	Rodentia	Rodent
**Biome_G_018**	LIB	4.915387042086516	-52.3613138909788	*Uranotaenia*	***Ur. (Ura.) nataliae***	FGMOS2905-22	*n.d.*		oui	*Rattus rattus*	5650	non	***Rattus Rattus***	Mammals	Rodentia	Rodent
**Biome_G_019**	LIB	4.915387042086516	-52.3613138909788	*Mansonia*	***Ma. (Man.) titillans***	MT999303.1	*Hydrochoerus hydrochaeris*	KX381515.1	oui	*Hydrochoerus hydrochaeris*	23454	non	***Hydrochoerus hydrochaeris***	Mammals	Rodentia	Rodent
**Biome_G_020**	HPF	4.9836667	-52.47788888888889	*Culex*	***Cx. (Mel.) eastor***	FGMOS2752-20	*n.d.*		oui	*Agouti paca*	156	oui	***Agouti paca***	Mammals	Rodentia	Rodent
**Biome_G_020**	HPF	4.9836667	-52.47788888888889	*Culex*	***Cx. (Mel.) eastor***	FGMOS2752-20	*n.d.*		/	*Zygodontomys brevicauda*	124	oui-m2	***Zygodontomys brevicauda***	Mammals	Rodentia	Rodent
**Biome_G_021**	HPF	4.9836667	-52.47788888888889	*Culex*	***Cx. (Mel.) eastor***	FGMOS2743-20	*n.d.*		non	Pas de minion	Pas de minion	/	***n.d.***	*n.d.*	*n.d.*	*n.d.*
**Biome_G_022**	HPF	4.9836667	-52.47788888888889	*Culex*	***Cx. (Mel.) eastor***	FGMOS2752-20	*Zygodontomys brevicauda*	KX381438.1	oui	*Zygodontomys brevicauda*	126	oui	***Zygodontomys brevicauda***	Mammals	Rodentia	Rodent
**Biome_G_022**	HPF	4.9836667	-52.47788888888889	*Culex*	***Cx. (Mel.) eastor***	FGMOS2752-20	*n.d.*		/	*Rattus rattus*	200	oui-m2	***Rattus Rattus***	Mammals	Rodentia	Rodent
**Biome_G_023**	HPF	4.9836667	-52.47788888888889	*Culex*	***n.d.***		N.T.		N.T.	N.T.	N.T.	N.T.	***N.T.***	N.T.	N.T.	N.T.
**Biome_G_024**	HPF	4.9836667	-52.47788888888889	*Culex*	***Cx. (Mel.) dunni***	FGMOS2748-20	*n.d.*		oui	*Rattus rattus*	6480	oui	***Rattus Rattus***	Mammals	Rodentia	Rodent
**Biome_G_024**	HPF	4.9836667	-52.47788888888889	*Culex*	***Cx. (Mel.) dunni***	FGMOS2748-20	*n.d.*		/	*Coendou prehensilis*	2269	oui-m2	***Coendou prehensilis***	Mammals	Rodentia	Rodent
**Biome_G_024**	HPF	4.9836667	-52.47788888888889	*Culex*	***Cx. (Mel.) dunni***	FGMOS2748-20	*n.d.*		/	*Zygodontomys brevicauda*	1784	oui-m3	***Zygodontomys brevicauda***	Mammals	Rodentia	Rodent
**Biome_G_025**	HPF	4.9836667	-52.47788888888889	*Culex*	***Cx. (Ads.) amazonensis***	FGMOS2756-20	*Agouti paca*	KX381423.1	oui	*Agouti paca*	5744	non	***Agouti paca***	Mammals	Rodentia	Rodent
**Biome_G_026**	HPF	4.9836667	-52.47788888888889	*Culex*	***Cx. (Mel.) portesi***	FGMOS2416-20	*Agouti paca*	KX381423.1	oui	*Agouti paca*	3216	non	***Agouti paca***	Mammals	Rodentia	Rodent
**Biome_G_027**	HPF	4.9836667	-52.47788888888889	*Culex*	***n.d.***		N.T.		N.T.	N.T.	N.T.	N.T.	***N.T.***	N.T.	N.T.	N.T.
**Biome_G_028**	HPF	4.9836667	-52.47788888888889	*Culex*	***n.d.***		N.T.		N.T.	N.T.	N.T.	N.T.	***N.T.***	N.T.	N.T.	N.T.
**Biome_G_029**	HPF	4.9836667	-52.47788888888889	*Culex*	***Cx. (Mel.) phlogistus***	FGMOS1541-20	*Agouti paca*	KX381423.1	oui	*Agouti paca*	1554	oui	***Agouti paca***	Mammals	Rodentia	Rodent
**Biome_G_029**	HPF	4.9836667	-52.47788888888889	*Culex*	***Cx. (Mel.) phlogistus***	FGMOS1541-20	*n.d.*		/	*Alouatta macconnelli*	1570	oui-m2	***Alouatta macconnelli***	Mammals	Primates	Non-human primate
**Biome_G_030**	HPF	4.9836667	-52.47788888888889	*Culex*	***Cx. (Mel.) rabelloi***	FGMOS2230-20	*Agouti paca*	KX381423.1	oui	*Agouti paca*	1440	non	***Agouti paca***	Mammals	Rodentia	Rodent
**Biome_G_031**	HPF	4.9836667	-52.47788888888889	*Culex*	***Cx. (Mel.) pedroi***	GBCAB35635-24	*Agouti paca*	KX381423.1	oui	*Agouti paca*	3376	non	***Agouti paca***	Mammals	Rodentia	Rodent
**Biome_G_032**	HPF	4.9836667	-52.47788888888889	*Culex*	***Cx. (Cux.) mollis***	FGMOS2271-20	*Agouti paca*	KX381423.1	oui	*Agouti paca*	4336	non	***Agouti paca***	Mammals	Rodentia	Rodent
**Biome_G_033**	HPF	4.9836667	-52.47788888888889	*Culex*	***Cx. (Cux.) quinquefasciatus***	FGMOS2276-20	*Agouti paca*	KX381423.1	oui	*Agouti paca*	174	oui	***Agouti paca***	Mammals	Rodentia	Rodent
**Biome_G_033**	HPF	4.9836667	-52.47788888888889	*Culex*	***Cx. (Cux.) quinquefasciatus***	FGMOS2276-20	*n.d.*		/	*Alouatta macconnelli*	300	oui-m2	***Alouatta macconnelli***	Mammals	Primates	Non-human primate
**Biome_G_034**	HPF	4.9836667	-52.47788888888889	*Culex*	***n.d.***		N.T.		N.T.	N.T.	N.T.	N.T.	***N.T.***	N.T.	N.T.	N.T.
**Biome_G_035**	HPF	4.9836667	-52.47788888888889	*Uranotaenia*	***n.d.***		N.T.		N.T.	N.T.	N.T.	N.T.	***N.T.***	N.T.	N.T.	N.T.
**Biome_G_036**	HPF	4.9836667	-52.47788888888889	*Uranotaenia*	***Ur. (Ura.) apicalis***	FGMOS3043-22	*Agouti paca*	KX381423.1	oui	*Agouti paca*	128	oui	***Agouti paca***	Mammals	Rodentia	Rodent
**Biome_G_036**	HPF	4.9836667	-52.47788888888889	*Uranotaenia*	***Ur. (Ura.) apicalis***	FGMOS3043-22	*n.d.*		/	*Alouatta macconnelli*	1830	oui-m2	***Alouatta macconnelli***	Mammals	Primates	Non-human primate
**Biome_G_037**	HPF	4.9836667	-52.47788888888889	*Culex*	***Cx. (Mel.) rabelloi***	FGMOS2881-22	*Agouti paca*	KX381423.1	oui	*Agouti paca*	182	non	***Agouti paca***	Mammals	Rodentia	Rodent
**Biome_G_038**	HPF	4.9836667	-52.47788888888889	*Culex*	***Cx. (Mel.) rabelloi***	FGMOS1717-20	*Rhinella margaritifera*	KF665019.1	oui	*Rhinella margaritifera*	4794	non	***Rhinella margaritifera***	Amphibians	Anura	Amphibian
**Biome_G_039**	HPF	4.9836667	-52.47788888888889	*Culex*	***Cx. (Mel.) alinkios***	FGMOS2194-20	*Kentropyx calcarata*	AY046458.1	non	Pas de minion	Pas de minion	/	***Kentropyx calcarata***	Squamates	*Squamata*	*Squamate*
**Biome_G_040**	HPF	4.9836667	-52.47788888888889	*Culex*	***Cx. (Mel.) eastor***	FGMOS2752-20	*n.d.*		non	Pas de minion	Pas de minion	/	***n.d.***	*n.d.*	*n.d.*	*n.d.*
**Biome_G_041**	HPF	4.9836667	-52.47788888888889	*Culex*	***Cx. (Mel.) rabelloi***	FGMOS2230-20	*Agouti paca*	KX381423.1	oui	*Agouti paca*	2740	non	***Agouti paca***	Mammals	Rodentia	Rodent
**Biome_G_042**	HPF	4.9836667	-52.47788888888889	*Culex*	***Cx. (Mel.) eastor***	FGMOS2752-20	*Agouti paca*	KX381423.1	oui	*Agouti paca*	3918	non	***Agouti paca***	Mammals	Rodentia	Rodent
**Biome_G_043**	HPF	4.9836667	-52.47788888888889	*Culex*	***Cx. (Mel.) contei***	FGMOS2742-20	*n.d.*		oui	*n.d.*	n.d.	***n.d.***	***n.d.***	*n.d.*	*n.d.*	*n.d.*
**Biome_G_044**	HPF	4.9836667	-52.47788888888889	*Culex*	***Cx. (Mel.) eastor***	FGMOS2743-20	*n.d.*		oui	*Agouti paca*	5720	oui	***Agouti paca***	Mammals	Rodentia	Rodent
**Biome_G_044**	HPF	4.9836667	-52.47788888888889	*Culex*	***Cx. (Mel.) eastor***	FGMOS2743-20	*n.d.*		/	*Bos taurus indicus*	7160	oui-m2	***Bos taurus indicus***	Mammals	Artiodactyla	Domestic cattle
**Biome_G_045**	HPF	4.9836667	-52.47788888888889	*Coquillettidia*	***Cq. (Rhy.) venezuelensis***	OP785627.1	*Dasypus kappleri*	KX381672.1	oui	*Dasypus kappleri*	5454	oui	***Dasypus kappleri***	Mammals	Cingulata	Armadillo
**Biome_G_045**	HPF	4.9836667	-52.47788888888889	*Coquillettidia*	***Cq. (Rhy.) venezuelensis***	OP785627.1	*n.d.*		/	*Agouti paca*	1718	oui-m2	***Agouti paca***	Mammals	Rodentia	Rodent

**Biome_G_046**	HPF	4.9836667	-52.477888888888889	*Culex*	***Cx. (Mel.) eastor***	FGMOS2743-20	*Agouti paca*	KX381423.1	oui	*Agouti paca*	1221	non	***Agouti paca***	Mammals	Rodentia	Rodent
**Biome_G_047**	HPF	4.9836667	-52.477888888888889	*Culex*	***Cx. (Mel.) pedroi***	GBCAB35634-24	*Agouti paca*	KX381423.1	oui	*Agouti paca*	3700	non	***Agouti paca***	Mammals	Rodentia	Rodent
**Biome_G_048**	HPF	4.9836667	-52.477888888888889	*Aedes*	***Ae. (Och.) taeniorhynchus***	MW339699.1	*Canis lupus familiaris*	OQ341164.1	oui	*Canis lupus familiaris*	9341	non	***Canis lupus familiaris***	Mammals	Carnivora	Dog
**Biome_G_049**	LIB	4.915387042086516	-52.3613138909788	*Culex*	***Cx. (Mel.) erraticus***	FGMOS3111-23	*Agouti paca*	KX381423.1	oui	*Agouti paca*	136	non	***Agouti paca***	Mammals	Rodentia	Rodent
**Biome_G_050**	LIB	4.915387042086516	-52.3613138909788	*Culex*	***Cx. (Mel.) clarki***	FGMOS3110-23	*n.d.*		oui	*Agouti paca*	8582	non	***Agouti paca***	Mammals	Rodentia	Rodent
**Biome_G_051**	LIB	4.915387042086516	-52.3613138909788	*Culex*	***Cx. (Mel.) erraticus***	FGMOS3111-23	*Agouti paca*	KX381423.1	oui	*Agouti paca*	5152	non	***Agouti paca***	Mammals	Rodentia	Rodent
**Biome_G_052**	LIB	4.915387042086516	-52.3613138909788	*Culex*	***Cx. (Mel.) rabanicolus***	FGMOS2768-20	*Agouti paca*	KX381423.1	oui	*Agouti paca*	6134	non	***Agouti paca***	Mammals	Rodentia	Rodent
**Biome_G_053**	LIB	4.915387042086516	-52.3613138909788	*Culex*	***Cx. (Mel.) erraticus***	FGMOS3111-23	*Agouti paca*	KX381423.1	oui	*Agouti paca*	1578	non	***Agouti paca***	Mammals	Rodentia	Rodent
**Biome_G_054**	DDC	4.853576607619447	-52.26967869892846	*Culex*	***Cx. (Mel.) portesi***	FGMOS2416-20	*Bradypus tridactylus*	KX381499.1	oui	*Bradypus tridactylus*	8474	non	***Bradypus tridactylus***	Mammals	Pilosa	Sloth
**Biome_G_055**	MAN	5.632552547405859	-53.66091898281266	*Mansonia*	***Ma. (Man.) titillans***	MT999303.1	*Agouti paca*	KX381423.1	oui	*Agouti paca*	2357	oui	***Agouti paca***	Mammals	Rodentia	Rodent
**Biome_G_055**	MAN	5.632552547405859	-53.66091898281266	*Mansonia*	***Ma. (Man.) titillans***	MT999303.1	*n.d.*		/	*Homo sapiens*	25850	oui-m2	***Homo sapiens***	Mammals	Primates	Human
**Biome_G_056**	MAN	5.632552547405859	-53.66091898281266	*Mansonia*	***Ma. (Man.) titillans***	MT999303.1	*Agouti paca*	KX381423.1	oui	*Agouti paca*	5742	oui	***Agouti paca***	Mammals	Rodentia	Rodent
**Biome_G_056**	MAN	5.632552547405859	-53.66091898281266	*Mansonia*	***Ma. (Man.) titillans***	MT999303.1	*n.d.*		/	*Homo sapiens*	684	oui-m2	***Homo sapiens***	Mammals	Primates	Human
**Biome_G_057**	MAN	5.632552547405859	-53.66091898281266	*Mansonia*	***Ma. (Man.) titillans***	MT999303.1	*Agouti paca*	KX381423.1	oui	*Agouti paca*	3162	non	***Agouti paca***	Mammals	Rodentia	Rodent
**Biome_G_058**	MAN	5.632552547405859	-53.66091898281266	*Mansonia*	***Ma. (Man.) titillans***	MT999303.1	*Agouti paca*	KX381423.1	oui	*Agouti paca*	851	non	***Agouti paca***	Mammals	Rodentia	Rodent
**Biome_G_059**	MAN	5.632552547405859	-53.66091898281266	*Mansonia*	***Ma. (Man.) titillans***	MT999303.1	*Agouti paca*	KX381423.1	oui	*Agouti paca*	382	oui	***Agouti paca***	Mammals	Rodentia	Rodent
**Biome_G_059**	MAN	5.632552547405859	-53.66091898281266	*Mansonia*	***Ma. (Man.) titillans***	MT999303.1	*n.d.*		/	*Alouatta macconnelli*	680	oui-m2	***Alouatta macconnelli***	Mammals	Primates	Non-human primate
**Biome_G_060**	MAN	5.632552547405859	-53.66091898281266	*Mansonia*	***Ma. (Man.) titillans***	MT999303.1	*Agouti paca*	KX381423.1	oui	*Agouti paca*	1359	non	***Agouti paca***	Mammals	Rodentia	Rodent
**Biome_G_061**	MAN	5.632552547405859	-53.66091898281266	*Mansonia*	***Ma. (Man.) titillans***	MT999303.1	*Agouti paca*	KX381423.1	oui	*Agouti paca*	4966	non	***Agouti paca***	Mammals	Rodentia	Rodent
**Biome_G_062**	MAN	5.632552547405859	-53.66091898281266	*Mansonia*	***Ma. (Man.) titillans***	MT999303.1	*Agouti paca*	KX381423.1	oui	*Agouti paca*	1518	oui	***Agouti paca***	Mammals	Rodentia	Rodent
**Biome_G_062**	MAN	5.632552547405859	-53.66091898281266	*Mansonia*	***Ma. (Man.) titillans***	MT999303.1	*n.d.*		/	*Homo sapiens*	1090	oui-m2	***Homo sapiens***	Mammals	Primates	Human
**Biome_G_063**	MAN	5.632552547405859	-53.66091898281266	*Mansonia*	***Ma. (Man.) titillans***	MT999303.1	*Agouti paca*	KX381423.1	oui	*Agouti paca*	5834	non	***Agouti paca***	Mammals	Rodentia	Rodent
**Biome_G_064**	MAN	5.632552547405859	-53.66091898281266	*Mansonia*	***Ma. (Man.) titillans***	MT999303.1	*Agouti paca*	KX381423.1	oui	*Agouti paca*	594	non	***Agouti paca***	Mammals	Rodentia	Rodent
**Biome_G_065**	MAN	5.632552547405859	-53.66091898281266	*Mansonia*	***Ma. (Man.) titillans***	MT999303.1	*Agouti paca*	KX381423.1	oui	*Agouti paca*	3788	oui	***Agouti paca***	Mammals	Rodentia	Rodent
**Biome_G_065**	MAN	5.632552547405859	-53.66091898281266	*Mansonia*	***Ma. (Man.) titillans***	MT999303.1	*n.d.*		/	*Alouatta macconnelli*	1640	oui-m2	***Alouatta macconnelli***	Mammals	Primates	Non-human primate
**Biome_G_066**	MAN	5.632552547405859	-53.66091898281266	*Mansonia*	***Ma. (Man.) titillans***	MT999303.1	*Agouti paca*	KX381423.1	oui	*Agouti paca*	3574	non	***Agouti paca***	Mammals	Rodentia	Rodent
**Biome_G_067**	MAN	5.632552547405859	-53.66091898281266	*Mansonia*	***Ma. (Man.) titillans***	MT999303.1	*n.d.*		oui	*Agouti paca*	5626	non	***Agouti paca***	Mammals	Rodentia	Rodent
**Biome_G_068**	MAN	5.632552547405859	-53.66091898281266	*Mansonia*	***Ma. (Man.) titillans***	MT999303.1	*Agouti paca*	KX381423.1	oui	n.d.	n.d.	n.d.	***Agouti paca***	Mammals	Rodentia	Rodent
**Biome_G_069**	MAN	5.632552547405859	-53.66091898281266	*Mansonia*	***Ma. (Man.) titillans***	MT999303.1	*Agouti paca*	KX381423.1	oui	*Agouti paca*	5822	oui	***Agouti paca***	Mammals	Rodentia	Rodent
**Biome_G_069**	MAN	5.632552547405859	-53.66091898281266	*Mansonia*	***Ma. (Man.) titillans***	MT999303.1	*n.d.*		/	*Alouatta macconnelli*	4370	oui-m2	***Alouatta macconnelli***	Mammals	Primates	Non-human primate
**Biome_G_070**	MAN	5.632552547405859	-53.66091898281266	*Mansonia*	***Ma. (Man.) titillans***	MT999303.1	*Agouti paca*	KX381423.1	oui	*Agouti paca*	5896	oui	***Agouti paca***	Mammals	Rodentia	Rodent
**Biome_G_070**	MAN	5.632552547405859	-53.66091898281266	*Mansonia*	***Ma. (Man.) titillans***	MT999303.1	*n.d.*		/	*Alouatta macconnelli*	570	oui-m2	***Alouatta macconnelli***	Mammals	Primates	Non-human primate
**Biome_G_071**	MAN	5.632552547405859	-53.66091898281266	*Mansonia*	***Ma. (Man.) titillans***	MT999303.1	*Agouti paca*	KX381423.1	non	Pas de minion	Pas de minion	/	***Agouti paca***	Mammals	Rodentia	Rodent
**Biome_G_072**	MAN	5.632552547405859	-53.66091898281266	*Mansonia*	***Ma. (Man.) titillans***	MT999303.1	*Agouti paca*	KX381423.	oui	*Agouti paca*	3344	non	***Agouti paca***	Mammals	Rodentia	Rodent
**Biome_G_073**	MAN	5.632552547405859	-53.66091898281266	*Mansonia*	***Ma. (Man.) titillans***	MT999303.1	*n.d.*		oui	*n.d.*	n.d.	n.d.	***n.d.***	*n.d.*	*n.d.*	*n.d.*
**Biome_G_074**	MAN	5.632552547405859	-53.66091898281266	*Mansonia*	***Ma. (Man.) titillans***	MT999303.1	*Dendrocygna autumnalis*	AF173717.1	oui	*n.d.*	n.d.	n.d.	***Dendrocygna autumnalis***	Birds	Anseriformes	Wild bird
**Biome_G_075**	MAN	5.632552547405859	-53.66091898281266	*Mansonia*	***Ma. (Man.) titillans***	MT999303.1	*n.d.*		oui	*n.d.*	n.d.	n.d.	***n.d.***	*n.d.*	*n.d.*	*n.d.*
**Biome_G_076**	MAN	5.632552547405859	-53.66091898281266	*Mansonia*	***Ma. (Man.) titillans***	MT999303.1	*n.d.*		oui	*n.d.*	n.d.	n.d.	***n.d.***	*n.d.*	*n.d.*	*n.d.*
**Biome_G_077**	MAN	5.632552547405859	-53.66091898281266	*Mansonia*	***Ma. (Man.) titillans***	MT999303.1	*n.d.*		oui	*n.d.*	n.d.	n.d.	***n.d.***	*n.d.*	*n.d.*	*n.d.*
**Biome_G_078**	MAN	5.632552547405859	-53.66091898281266	*Mansonia*	***Ma. (Man.) titillans***	MT999303.1	*n.d.*		oui	*n.d.*	n.d.	n.d.	***n.d.***	*n.d.*	*n.d.*	*n.d.*
**Biome_G_079**	MAN	5.632552547405859	-53.66091898281266	*Mansonia*	***Ma. (Man.) titillans***	MT999303.1	*n.d.*		oui	*Homo sapiens*	42	non	***Homo sapiens***	Mammals	Primates	Human
**Biome_G_080**	MAN	5.632552547405859	-53.66091898281266	*Mansonia*	***Ma. (Man.) titillans***	MT999303.1	*Rattus rattus*	KX381445.1	oui	*Rattus rattus*	58980	non	***Rattus Rattus***	Mammals	Rodentia	Rodent
**Biome_G_081**	HPF	4.9836667	-52.477888888888889	*Coquillettidia*	***Cq. (Rhy.) venezuelensis***	OM630650.1	*Dasypus guianensis*	PP916004.1	oui	*Dasypus guianensis*	1259	non	***Dasypus guianensis***	Mammals	Cingulata	Armadillo
**Biome_G_082**	HPF	4.9836667	-52.477888888888889	*Coquillettidia*	***Cq. (Rhy.) venezuelensis***	OP785627.1	*n.d.*		oui	*Proechimys guyannensis*	624	non	***Proechimys guyannensis***	Mammals	Rodentia	Rodent
**Biome_G_083**	HPF	4.9836667	-52.477888888888889	*Culex*	***Cx. (Mel.) phlogistus***	FGMOS3175-23	*n.d.*		oui	*n.d.*	n.d.	n.d.	***n.d.***	*n.d.*	*n.d.*	*n.d.*
**Biome_G_084**	HPF	4.9836667	-52.477888888888889	*Culex*	***Cx. (Mel.) portesi***	FGMOS2416-20	*n.d.*		oui	*n.d.*	n.d.	n.d.	***n.d.***	*n.d.*	*n.d.*	*n.d.*
**Biome_G_085**	HPF	4.9836667	-52.477888888888889	*Culex*	***Cx. (Mel.) dunni***	FGMOS2751-20	*n.d.*		oui	*Agouti paca*	6460	non	***Agouti paca***	Mammals	Rodentia	Rodent
**Biome_G_086**	HPF	4.9836667	-52.477888888888889	*Culex*	***Cx. (Mel.) zeteki***	FGMOS2156-20	*n.d.*		oui	*n.d.*	n.d.	n.d.	***n.d.***	*n.d.*	*n.d.*	*n.d.*
**Biome_G_087**	HPF	4.9836667	-52.477888888888889	*Culex*	***Cx. (Mel.) dunni***	FGMOS2748-20	*Coendou prehensilis*	KX381447.1	oui	*Coendou prehensilis*	680	oui	***Coendou prehensilis***	Mammals	Rodentia	Rodent
**Biome_G_087**	HPF	4.9836667	-52.477888888888889	*Culex*	***Cx. (Mel.) dunni***	FGMOS2748-20	*n.d.*		/	*Homo sapiens*	820	oui-m2	***Homo sapiens***	Mammals	Primates	Human
**Biome_G_087**	HPF	4.9836667	-52.477888888888889	*Culex*	***Cx. (Mel.) dunni***	FGMOS2748-20	*n.d.*		/	*Zygodontomys brevicauda*	224	oui-m3	***Zygodontomys brevicauda***	Mammals	Rodentia	Rodent
**Biome_G_088**	HPF	4.9836667	-52.477888888888889	*Culex*	***Cx. (Mel.) dunni***	FGMOS2748-20	*Canis lupus familiaris*	OQ341164.1	oui	*Canis lupus familiaris*	5684	non	***Canis lupus familiaris***	Mammals	Carnivora	Dog
**Biome_G_089**	HPF	4.9836667	-52.477888888888889	*Uranotaenia*	***Ur. (Ura.) apicalis***	FGMOS3043-22	*n.d.*		oui	*n.d.*	n.d.	n.d.	***n.d.***	*n.d.*	*n.d.*	*n.d.*
**Biome_G_090**	HPF	4.9836667	-52.477888888888889	*Uranotaenia*	***n.d.***		N.T.		N.T.	N.T.	N.T.	N.T.	***N.T.***	N.T.	N.T.	N.T.
**Biome_G_091**	HPF	4.9836667	-52.477888888888889	*Uranotaenia*	***n.d.***		N.T.		N.T.	N.T.	N.T.	N.T.	***N.T.***	N.T.	N.T.	N.T.
**Biome_G_092**	HPF	4.9836667	-52.477888888888889	*Culex*	***Cx. (Mel.) phlogistus***	FGMOS1542-20	*Proechimys guyannensis*	KX755316.1	oui	*Proechimys guyannensis*	246	non	***Proechimys guyannensis***	Mammals	Rodentia	Rodent
**Biome_G_093**	HPF	4.9836667	-52.477888888888889	*Culex*	***n.d.***		N.T.		N.T.	N.T.	N.T.	N.T.	***N.T.***	N.T.	N.T.	N.T.
**Biome_G_094**	HPF	4.9836667	-52.477888888888889	*Coquillettidia*	***Cq. (Rhy.) venezuelensis***	OM630650.1	*Dasypus guianensis*	PP916004.1	oui	*Dasypus guianensis*	1104	non	***Dasypus guianensis***	Mammals	Cingulata	Armadillo
**Biome_G_095**	HPF	4.9836667	-52.477888888888889	*Coquillettidia*	***Cq. (Rhy.) venezuelensis***	OP785627.1	*n.d.*		oui	*Dasypus guianensis*	48663	non	***Dasypus guianensis***	Mammals	Cingulata	Armadillo
**Biome_G_096**	HPF	4.9836667	-52.477888888888889	*Coquillettidia*	***Cq. (Rhy.) venezuelensis***	OM630650.1	*Dasypus guianensis*	PP916004.1	oui	*Dasypus guianensis*	817	non	***Dasypus guianensis***	Mammals	Cingulata	Armadillo
**Biome_G_097**	HPF	4.9836667	-52.477888888888889	*Culex*	***Cx. (Mel.) portesi***	FGMOS2416-20	*n.d.*		oui	*Proechimys guyannensis*	3108	non	***Proechimys guyannensis***	Mammals	Rodentia	Rodent
**Biome_G_098**	HPF	4.9836667	-52.477888888888889	*Aedes*	***Ae. (Och.) taeniorhynchus***	MW339699.1	*Bos taurus indicus*	OP859012.1	non	Pas de minion	Pas de minion	/	***Bos taurus indicus***	Mammals	Artiodactyla	Domestic cattle
**Biome_G_099**	HPF	4.9836667	-52.477888888888889	*Culex*	***Cx. (Cux.) declarator***	FGMOS2281-20	*Coturnix coturnix*	KF469290.1	oui	*Coturnix coturnix*	2008	non	***Coturnix coturnix***	Birds	Galliformes	Domestic bird
**Biome_G_100**	HPF	4.9836667	-52.477888888888889	*Culex*	***Cx. (Cux.) nigripalpus***	FGMOS2667-20	*Thamnophilus nigrocinereus*	KJ909192.1	oui	*Thamnophilus nigrocinereus*	505	non	***Thamnophilus nigrocinereus***	Birds	Passeriformes	Wild bird
**Biome_G_101**	HPF	4.9836667	-52.477888888888889	*Culex*	***Cx. (Mel.) portesi***	FGMOS2416-20	*Bos taurus indicus*	OP859012.1	oui	*Bos taurus indicus*	8870	non	***Bos taurus indicus***	Mammals	Artiodactyla	Domestic cattle
**Biome_G_102**	HPF	4.9836667	-52.477888888888889	*Culex*	***Cx. (Cux.) nigripalpus***	FGMOS2667-20	*Coendou prehensilis*	KX381447.1	oui	*Coendou prehensilis*	200	non	***Coendou prehensilis***	Mammals	Rodentia	Rodent
**Biome_G_103**	HPF	4.9836667	-52.477888888888889	*Culex*	***Cx. (Mel.) contei***	FGMOS2742-20	*Epicrates cenchria*	AF368059.1	oui	*Epicrates cenchria*	1869	non	***Epicrates cenchria***	Squamates	Squamata	Squamate
**Biome_G_104**	HPF	4.9836667	-52.477888888888889	*Culex*	***Cx. (Ads.) amazonensis***	FGMOS2761-20	*Didelphis marsupialis*	KX381440.1	oui	*Didelphis marsupialis*	401	oui	***Didelphis marsupialis***	Mammals	Didelphimorphia	Marsupial
**Biome_G_104**	HPF	4.9836667	-52.477888888888889	*Culex*	***Cx. (Ads.) amazonensis***	FGMOS2761-20	*n.d.*		/	*Metachirus nudicaudatus*	58	oui-m2	***Metachirus nudicaudatus***	Mammals	Didelphimorphia	Marsupial
**Biome_G_105**	HPF	4.9836667	-52.477888888888889	*Culex*	***Cx. (Mel.) portesi***	FGMOS2416-20	*n.d.*		non	Pas de minion	Pas de minion	/	***n.d.***	*n.d.*	*n.d.*	*n.d.*
**Biome_G_106**	HPF	4.9836667	-52.477888888888889	*Culex*	***Cx. (Mel.) portesi***	FGMOS2416-20	*n.d.*		oui	*n.d.*	n.d.	n.d.	***n.d.***	*n.d.*	*n.d.*	*n.d.*
**Biome_G_107**	HPF	4.9836667	-52.477888888888889	*Culex*	***Cx. (Mel.) portesi***	FGMOS2416-20	*n.d.*		non	Pas de minion	Pas de minion	/	***n.d.***	*n.d.*	*n.d.*	*n.d.*

Biome_G_108	PAL	4.051826178135371	-51.66942020699708	Culex	Cx. (Mel.) portesi	FGMOS2416-20	Hylaeamys megacephalus	KX381569.1	non	Pas de minion	Pas de minion	/	Hylaeamys megacephalus	Mammals	Rodentia	Rodent
Biome_G_109	PAL	4.051826178135371	-51.66942020699708	Culex	Cx. (Mel.) portesi	FGMOS2416-20	Philander opossum	KX381605.1	oui	Philander opossum	2624	non	Philander opossum	Mammals	Didelphimorphia	Marsupial
Biome_G_110	PAL	4.051826178135371	-51.66942020699708	Culex	Cx. (Mel.) dunni	FGMOS2416-20	Gallus gallus	OQ562002.1	oui	n.d.	630	n.d.	Gallus gallus	Birds	Galliformes	Domestic bird
Biome_G_111	PAL	4.051826178135371	-51.66942020699708	Culex	Cx. (Mel.) pedroi	FGMOS2748-20	Homo sapiens	MF437201.1	oui	Homo sapiens	163820	non	Homo sapiens	Mammals	Primates	Human
Biome_G_112	PAL	4.051826178135371	-51.66942020699708	Culex	n.d.		N.T.		N.T.	N.T.	N.T.	N.T.	N.T.	N.T.	N.T.	N.T.
Biome_G_113	PAL	4.051826178135371	-51.66942020699708	Culex	Cx. (Mel.) phlogistus	FGMOS3175-23	Rhinella marina	KF664764.1	oui	Rhinella marina	3500	non	Rhinella marina	Amphibians	Anura	Amphibian
Biome_G_114	PAL	4.051826178135371	-51.66942020699708	Culex	Cx. (Mel.) phlogistus	FGMOS3175-23	Philander opossum	KX381605.1	oui	Philander opossum	10594	non	Philander opossum	Mammals	Didelphimorphia	Marsupial
Biome_G_115	PAL	4.051826178135371	-51.66942020699708	Culex	Cx. (Mel.) portesi	FGMOS2416-20	Marmosa demerarae	KX381531.1	oui	Marmosa demerarae	3195	non	Marmosa demerarae	Mammals	Didelphimorphia	Marsupial
Biome_G_116	HPF	4.9836667	-52.47788888888889	Culex	Cx. (Mel.) portesi	FGMOS2416-20	n.d.		oui	Oecomys bicolor	66	non	Oecomys bicolor	Mammals	Rodentia	Rodent
Biome_G_117	HPF	4.9836667	-52.47788888888889	Culex	Cx. (Mel.) eastor	FGMOS2743-20	Oecomys bicolor	KX381448.1	oui	Oecomys bicolor	850	non	Oecomys bicolor	Mammals	Rodentia	Rodent
Biome_G_118	HPF	4.9836667	-52.47788888888889	Culex	Cx. (Mel.) pedroi	GBCAB35634-24	Gallus gallus	OQ562002.1	oui	n.d.	n.d.	n.d.	Gallus gallus	Birds	Galliformes	Domestic bird
Biome_G_119	HPF	4.9836667	-52.47788888888889	Culex	n.d.		N.T.		N.T.	N.T.	N.T.	N.T.	N.T.	N.T.	N.T.	N.T.
Biome_G_120	HPF	4.9836667	-52.47788888888889	Culex	Cx. (Mel.) dunni	FGMOS2748-20	n.d.		oui	n.d.	n.d.	n.d.	n.d.	n.d.	n.d.	n.d.
Biome_G_121	HPF	4.9836667	-52.47788888888889	Culex	Cx. (Mel.) spissipes	FGMOS2701-20	n.d.		oui	Homo sapiens	4830	non	Homo sapiens	Mammals	Primates	Human
Biome_G_122	HPF	4.9836667	-52.47788888888889	Culex	Cx. (Ads.) amazonensis	FGMOS2761-20	n.d.		oui	n.d.	n.d.	n.d.	n.d.	n.d.	n.d.	n.d.
Biome_G_123	ROU	4.741465576104164	-52.325689611546075	Culex	Cx. (Mel.) ernsti	FGMOS3102-23	n.d.		oui	Homo sapiens	640	oui	Homo sapiens	Mammals	Primates	Human
Biome_G_123	ROU	4.741465576104164	-52.325689611546075	Culex	Cx. (Mel.) ernsti	FGMOS3102-23	n.d.		/	Zygodontomys brevicauda	238	oui-m2	Zygodontomys brevicauda	Mammals	Rodentia	Rodent
Biome_G_124	ROU	4.741465576104164	-52.325689611546075	Culex	Cx. (Mel.) portesi	FGMOS2416-20	Dasypus guianensis	PP916004.1	oui	Dasypus guianensis	13038	non	Dasypus guianensis	Mammals	Cingulata	Armadillo
Biome_G_125	ROU	4.741465576104164	-52.325689611546075	Culex	Cx. (Mel.) portesi	FGMOS2416-20	Choloepus didactylus	KX779919.1	oui	Choloepus didactylus	4912	non	Choloepus didactylus	Mammals	Pilosa	Sloth
Biome_G_126	ROU	4.741465576104164	-52.325689611546075	Culex	Cx. (Mel.) spissipes	FGMOS2701-20	Chironectes minimus	KX381736.1	oui	n.d.	n.d.	n.d.	Chironectes minimus	Mammals	Didelphimorphia	Marsupial
Biome_G_127	HPF	4.9836667	-52.47788888888889	Culex	Cx. (Mel.) pedroi	GBCAB35634-24	n.d.		oui	n.d.	n.d.	n.d.	n.d.	n.d.	n.d.	n.d.
Biome_G_128	HPF	4.9836667	-52.47788888888889	Culex	Cx. (Mel.) pedroi	GBCAB35634-24	Thamnophilus nigrocinereus	KJ909192.1	oui	Thamnophilus nigrocinereus	357	non	Thamnophilus nigrocinereus	Birds	Passeriformes	Wild bird
Biome_G_129	HPF	4.9836667	-52.47788888888889	Culex	Cx. (Mel.) portesi	FGMOS2416-20	Homo sapiens	MK069563.1	oui	Homo sapiens	24110	non	Homo sapiens	Mammals	Primates	Human
Biome_G_130	HPF	4.9836667	-52.47788888888889	Culex	Cx. (Cux.) nigripalpus	FGMOS2667-20	Homo sapiens	MF437201.1	oui	Homo sapiens	2040	non	Homo sapiens	Mammals	Primates	Human
Biome_G_131	HPF	4.9836667	-52.47788888888889	Culex	Cx. (Cux.) declarator	FGMOS2750-20	n.d.		non	Pas de minion	Pas de minion	/	n.d.	n.d.	n.d.	n.d.
Biome_G_132	HPF	4.9836667	-52.47788888888889	Culex	Cx. (Mel.) dunni	FGMOS2750-20	n.d.		oui	n.d.	n.d.	n.d.	n.d.	n.d.	n.d.	n.d.
Biome_G_133	HPF	4.9836667	-52.47788888888889	Culex	Cx. (Cux.) nigripalpus	FGMOS2667-20	n.d.		non	Pas de minion	Pas de minion	/	n.d.	n.d.	n.d.	n.d.
Biome_G_134	HPF	4.9836667	-52.47788888888889	Culex	Cx. (Mel.) dunni	FGMOS2748-20	n.d.		oui	Didelphis marsupialis	5826	oui	Didelphis marsupialis	Mammals	Didelphimorphia	Marsupial
Biome_G_134	HPF	4.9836667	-52.47788888888889	Culex	Cx. (Mel.) dunni	FGMOS2748-20	n.d.		/	Metachirus nudicaudatus	1942	oui-m2	Metachirus nudicaudatus	Mammals	Didelphimorphia	Marsupial
Biome_G_135	HPF	4.9836667	-52.47788888888889	Culex	Cx. (Cux.) nigripalpus	FGMOS2667-20	Homo sapiens	PP999313.1	oui	n.d.	n.d.	n.d.	Homo sapiens	Mammals	Primates	Human
Biome_G_136	HPF	4.9836667	-52.47788888888889	Culex	Cx. (Mel.) pedroi	GBCAB35634-24	n.d.		oui	Dasyprocta leporina	3381	non	Dasyprocta leporina	Mammals	Rodentia	Rodent
Biome_G_137	HPF	4.9836667	-52.47788888888889	Culex	Cx. (Cux.) nigripalpus	FGMOS2667-20	n.d.		oui	n.d.	n.d.	n.d.	n.d.	n.d.	n.d.	n.d.
Biome_G_138	HPF	4.9836667	-52.47788888888889	Culex	Cx. (Mel.) dunni	FGMOS2748-20	n.d.		oui	Sakesphorus luctuosus	579	non	Sakesphorus luctuosus	Birds	Passeriformes	Wild bird
Biome_G_139	HPF	4.9836667	-52.47788888888889	Culex	Cx. (Mel.) eastor	FGMOS2695-20	Homo sapiens	MN848587.1	oui	n.d.	n.d.	n.d.	Homo sapiens	Mammals	Primates	Human
Biome_G_140	HPF	4.9836667	-52.47788888888889	Culex	Cx. (Mel.) dunni	FGMOS2748-20	Trogon viridis	U94817.1	non	Pas de minion	Pas de minion	/	Trogon viridis	Birds	Trogoniformes	Wild bird
Biome_G_141	HPF	4.9836667	-52.47788888888889	Culex	Cx. (Mel.) contei	FGMOS2742-20	n.d.		non	Pas de minion	Pas de minion	/	n.d.	n.d.	n.d.	n.d.
Biome_G_142	HPF	4.9836667	-52.47788888888889	Culex	Cx. (Mel.) dunni	FGMOS2156-20	n.d.		oui	n.d.	n.d.	n.d.	n.d.	n.d.	n.d.	n.d.
Biome_G_143	HPF	4.9836667	-52.47788888888889	Culex	Cx. (Mel.) dunni	FGMOS2748-20	Coendou prehensilis	KX381447.1	non	Pas de minion	Pas de minion	/	Coendou prehensilis	Mammals	Rodentia	Rodent
Biome_G_144	HPF	4.9836667	-52.47788888888889	Culex	Cx. (Mel.) dunni	FGMOS2750-20	n.d.		oui	n.d.	n.d.	n.d.	n.d.	n.d.	n.d.	n.d.
Biome_G_145	PAL	4.051826178135371	-51.66942020699708	Coquillettidia	Cq. (Rhy.) albicosta	OP785646.1	Pteronura brasiliensis	KX381453.1	non	Pas de minion	Pas de minion	/	Pteronura brasiliensis	Mammals	Carnivora	Otter
Biome_G_146	PAL	4.051826178135371	-51.66942020699708	Culex	Cx. (Mel.) dunni	FGMOS2748-20	n.d.		oui	Canis lupus familiaris	22914	non	Canis lupus familiaris	Mammals	Carnivora	Dog
Biome_G_147	PAL	4.051826178135371	-51.66942020699708	Limatus	n.d.		N.T.		N.T.	N.T.	N.T.	N.T.	N.T.	N.T.	N.T.	N.T.
Biome_G_148	HPF	4.9836667	-52.47788888888889	Culex	Cx. (Mel.) portesi	FGMOS2416-20	n.d.		non	Pas de minion	Pas de minion	/	n.d.	n.d.	n.d.	n.d.
Biome_G_149	HPF	4.9836667	-52.47788888888889	Limatus	Li. Durhamii	MF172330.1	n.d.		non	Pas de minion	Pas de minion	/	n.d.	n.d.	n.d.	n.d.
Biome_G_150	HPF	4.9836667	-52.47788888888889	Aedes	Ae. (Och.) taeniorhynchus	MW339699.1	n.d.		non	Pas de minion	Pas de minion	/	n.d.	n.d.	n.d.	n.d.
Biome_G_151	HPF	4.9836667	-52.47788888888889	Culex	Cx. (Mel.) zeteki	FGMOS2156-20	n.d.		non	Pas de minion	Pas de minion	/	n.d.	n.d.	n.d.	n.d.
Biome_G_152	HPF	4.9836667	-52.47788888888889	Culex	Cx. (Mel.) zeteki	FGMOS2156-20	n.d.		oui	n.d.	n.d.	n.d.	n.d.	n.d.	n.d.	n.d.
Biome_G_153	HPF	4.9836667	-52.47788888888889	Culex	Cx. (Mel.) zeteki	FGMOS2156-20	n.d.		non	Pas de minion	Pas de minion	/	n.d.	n.d.	n.d.	n.d.
Biome_G_154	HPF	4.9836667	-52.47788888888889	Culex	Cx. (Mel.) dunni	FGMOS2748-20	Gallus gallus	OR834462.1	non	Pas de minion	Pas de minion	/	Gallus gallus	Birds	Galliformes	Domestic bird
Biome_G_155	HPF	4.9836667	-52.47788888888889	Aedes	n.d.		N.T.		N.T.	N.T.	N.T.	N.T.	N.T.	N.T.	N.T.	N.T.
Biome_G_156	HPF	4.9836667	-52.47788888888889	Aedes	n.d.		N.T.		N.T.	N.T.	N.T.	N.T.	N.T.	N.T.	N.T.	N.T.
Biome_G_157	HPF	4.9836667	-52.47788888888889	Coquillettidia	Cq. (Rhy.) venezuelensis	OM630650.1	Dasypus guianensis	PP916004.1	non	Pas de minion	Pas de minion	/	Dasypus guianensis	Mammals	Cingulata	Armadillo
Biome_G_158	HPF	4.9836667	-52.47788888888889	Culex	Cx. (Mel.) eastor	FGMOS2743-20	n.d.		oui	Homo sapiens	710	non	Homo sapiens	Mammals	Primates	Human
Biome_G_159	HPF	4.9836667	-52.47788888888889	Culex	Cx. (Cux.) nigripalpus	FGMOS2667-20	n.d.		oui	n.d.	n.d.	n.d.	n.d.	n.d.	n.d.	n.d.
Biome_G_160	HPF	4.9836667	-52.47788888888889	Culex	Cx. (Cux.) nigripalpus	FGMOS2667-20	n.d.		non	Pas de minion	Pas de minion	/	n.d.	n.d.	n.d.	n.d.
Biome_G_161	HPF	4.9836667	-52.47788888888889	Culex	Cx. (Cux.) nigripalpus	FGMOS2667-20	n.d.		non	Pas de minion	Pas de minion	/	n.d.	n.d.	n.d.	n.d.
Biome_G_162	HPF	4.9836667	-52.47788888888889	Culex	Cx. (Mel.) dunni	FGMOS2748-20	n.d.		oui	n.d.	n.d.	n.d.	n.d.	n.d.	n.d.	n.d.
Biome_G_163	HPF	4.9836667	-52.47788888888889	Culex	Cx. (Mel.) zeteki	FGMOS2156-20	n.d.		non	Pas de minion	Pas de minion	/	n.d.	n.d.	n.d.	n.d.
Biome_G_164	HPF	4.9836667	-52.47788888888889	Culex	Cx. (Mel.) dunni	FGMOS2748-20	Thamnophilus nigrocinereus	KJ909192.1	oui	Thamnophilus nigrocinereus	3484	non	Thamnophilus nigrocinereus	Birds	Passeriformes	Wild bird
Biome_G_165	HPF	4.9836667	-52.47788888888889	Culex	Cx. (Mel.) zeteki	FGMOS2156-20	n.d.		non	Pas de minion	Pas de minion	/	n.d.	n.d.	n.d.	n.d.
Biome_G_166	HPF	4.9836667	-52.47788888888889	Culex	Cx. (Mel.) dunni	FGMOS2748-20	Coendou prehensilis	KX381447.1	oui	Coendou prehensilis	7820	non	Coendou prehensilis	Mammals	Rodentia	Rodent
Biome_G_167	HPF	4.9836667	-52.47788888888889	Coquillettidia	Cq. (Rhy.) venezuelensis	OM630650.1	Agouti paca	KX381423.1	oui	Agouti paca	7716	non	Agouti paca	Mammals	Rodentia	Rodent
Biome_G_168	HPF	4.9836667	-52.47788888888889	Coquillettidia	Cq. (Rhy.) venezuelensis	OP785627.1	Choloepus didactylus	KX779919.1	oui	Choloepus didactylus	11648	non	Choloepus didactylus	Mammals	Pilosa	Sloth
Biome_G_169	HPF	4.9836667	-52.47788888888889	Coquillettidia	Cq. (Rhy.) venezuelensis	OM630650.1	n.d.		oui	n.d.	n.d.	n.d.	n.d.	n.d.	n.d.	n.d.
Biome_G_170	HPF	4.9836667	-52.47788888888889	Coquillettidia	Cq. (Rhy.) venezuelensis	OM630650.1	Homo sapiens	MF437201.1	oui	n.d.	n.d.	n.d.	Homo sapiens	Mammals	Primates	Human
Biome_G_171	HPF	4.9836667	-52.47788888888889	Culex	Cx. (Mel.) eastor	FGMOS2743-20	n.d.		oui	n.d.	n.d.	n.d.	n.d.	n.d.	n.d.	n.d.
Biome_G_172	HPF	4.9836667	-52.47788888888889	Culex	Cx. (Mel.) rabanicolus	FGMOS2744-20	n.d.		oui	n.d.	n.d.	n.d.	n.d.	n.d.	n.d.	n.d.
Biome_G_173	HPF	4.9836667	-52.47788888888889	Culex	Cx. (Mel.) phlogistus	FGMOS1542-20	n.d.		oui	n.d.	n.d.	n.d.	n.d.	n.d.	n.d.	n.d.
Biome_G_174	HPF	4.9836667	-52.47788888888889	Culex	Cx. (Mel.) zeteki	FGMOS2156-20	n.d.		oui	n.d.	n.d.	n.d.	n.d.	n.d.	n.d.	n.d.
Biome_G_175	HPF	4.9836667	-52.47788888888889	Culex	Cx. (Mel.) rabanicolus	FGMOS2768-20	n.d.		oui	n.d.	n.d.	n.d.	n.d.	n.d.	n.d.	n.d.
Biome_G_176	HPF	4.9836667	-52.47788888888889	Culex	Cx. (Mel.) zeteki	FGMOS2156-20	n.d.		oui	n.d.	n.d.	n.d.	n.d.	n.d.	n.d.	n.d.
Biome_G_177	HPF	4.9836667	-52.47788888888889	Culex	Cx. (Mel.) phlogistus	FGMOS3175-23	n.d.		oui	n.d.	n.d.	n.d.	n.d.	n.d.	n.d.	n.d.

**Biome_G_178**	HPF	4.9836667	-52.47788888888889	*Culex*	***Cx. (Mel.) phlogistus***	FGMOS1542-20	*n.d.*		oui	*n.d.*	n.d.	n.d.	***n.d.***	*n.d.*	*n.d.*	*n.d.*
**Biome_G_179**	HPF	4.9836667	-52.47788888888889	*Culex*	***Cx. (Mel.) eastor***	FGMOS2743-20	*n.d.*		oui	*n.d.*	n.d.	n.d.	***n.d.***	*n.d.*	*n.d.*	*n.d.*
**Biome_G_180**	HPF	4.9836667	-52.47788888888889	*Culex*	***Cx. (Mel.) eastor***	FGMOS2743-20	*n.d.*		oui	*Homo sapiens*	56020	non	***Homo sapiens***	Mammals	Primates	Human
**Biome_G_181**	HPF	4.9836667	-52.47788888888889	*Culex*	***Cx. (Mel.) phlogistus***	FGMOS3175-23	*Gonatodes humeralis*	GU139942.1	non	Pas de minion	Pas de minion	/	***Gonatodes humeralis***	Squamates	Squamata	Squamate
**Biome_G_182**	HPF	4.9836667	-52.47788888888889	*Culex*	***Cx. (Mel.) eastor***	FGMOS2752-20	*n.d.*		oui	*n.d.*	n.d.	n.d.	***n.d.***	*n.d.*	*n.d.*	*n.d.*
**Biome_G_183**	HPF	4.9836667	-52.47788888888889	*Culex*	***Cx. (Mel.) eastor***	FGMOS2752-20	*Bubalus bubalis*	MT253542.1	oui	*Bubalus bubalis*	44870	non	***Bubalus bubalis***	Mammals	Artiodactyla	Domestic cattle
**Biome_G_184**	HPF	4.9836667	-52.47788888888889	*Culex*	***Cx. (Mel.) eastor***	FGMOS2752-20	*n.d.*		oui	*n.d.*	n.d.	n.d.	***n.d.***	*n.d.*	*n.d.*	*n.d.*
**Biome_G_185**	HPF	4.9836667	-52.47788888888889	*Culex*	***n.d.***		N.T.		N.T.	N.T.	N.T.	N.T.	***N.T.***	N.T.	N.T.	N.T.
**Biome_G_186**	HPF	4.9836667	-52.47788888888889	*Culex*	***Cx. (Mel.) phlogistus***	FGMOS1542-20	*Gonatodes humeralis*	GU139942.1	non	Pas de minion	Pas de minion	/	***Gonatodes humeralis***	Squamates	Squamata	Squamate
**Biome_G_187**	HPF	4.9836667	-52.47788888888889	*Culex*	***Cx. (Mel.) pedroi***	GBCAB35634-24	*n.d.*		oui	*n.d.*	n.d.	n.d.	***n.d.***	*n.d.*	*n.d.*	*n.d.*
**Biome_G_188**	HPF	4.9836667	-52.47788888888889	*Culex*	***Cx. (Mel.) contei***	FGMOS2742-20	*Gonatodes humeralis*	GU139942.1	non	Pas de minion	Pas de minion	/	***Gonatodes humeralis***	Squamates	Squamata	Squamate
**Biome_G_189**	HPF	4.9836667	-52.47788888888889	*Culex*	***Cx. (Mel.) pedroi***	GBCAB35634-24	*n.d.*		oui	*n.d.*	n.d.	n.d.	***n.d.***	*n.d.*	*n.d.*	*n.d.*
**Biome_G_190**	HPF	4.9836667	-52.47788888888889	*Culex*	***n.d.***		N.T.		N.T.	N.T.	N.T.	N.T.	***N.T.***	N.T.	N.T.	N.T.
**Biome_G_191**	HPF	4.9836667	-52.47788888888889	*Culex*	***Cx. (Mel.) phlogistus***	FGMOS3175-23	*Gonatodes humeralis*	GU139942.1	oui	*Gonatodes humeralis*	1483	non	***Gonatodes humeralis***	Squamates	Squamata	Squamate
**Biome_G_192**	HPF	4.9836667	-52.47788888888889	*Culex*	***Cx. (Mel.) spissipes***	FGMOS2701-20	*n.d.*		non	Pas de minion	Pas de minion	/	***n.d.***	*n.d.*	*n.d.*	*n.d.*
**Biome_G_193**	MAN	5.632552547405859	-53.66091898281266	*Mansonia*	***n.d.***		N.T.		N.T.	N.T.	N.T.	N.T.	***N.T.***	N.T.	N.T.	N.T.
**Biome_G_194**	MAN	5.632552547405859	-53.66091898281266	*Mansonia*	***n.d.***		N.T.		N.T.	N.T.	N.T.	N.T.	***N.T.***	N.T.	N.T.	N.T.
**Biome_G_195**	MAN	5.632552547405859	-53.66091898281266	*Mansonia*	***n.d.***		N.T.		N.T.	N.T.	N.T.	N.T.	***N.T.***	N.T.	N.T.	N.T.
**Biome_G_196**	MAN	5.632552547405859	-53.66091898281266	*Mansonia*	***n.d.***		N.T.		N.T.	N.T.	N.T.	N.T.	***N.T.***	N.T.	N.T.	N.T.
**Biome_G_197**	MAN	5.632552547405859	-53.66091898281266	*Mansonia*	***n.d.***		N.T.		N.T.	N.T.	N.T.	N.T.	***N.T.***	N.T.	N.T.	N.T.
**Biome_G_198**	MAN	5.632552547405859	-53.66091898281266	*Mansonia*	***n.d.***		N.T.		N.T.	N.T.	N.T.	N.T.	***N.T.***	N.T.	N.T.	N.T.
**Biome_G_199**	MAN	5.632552547405859	-53.66091898281266	*Mansonia*	***n.d.***		N.T.		N.T.	N.T.	N.T.	N.T.	***N.T.***	N.T.	N.T.	N.T.
**Biome_G_200**	MAN	5.632552547405859	-53.66091898281266	*Mansonia*	***n.d.***		N.T.		N.T.	N.T.	N.T.	N.T.	***N.T.***	N.T.	N.T.	N.T.
**Biome_G_201**	MAN	5.632552547405859	-53.66091898281266	*Mansonia*	***n.d.***		N.T.		N.T.	N.T.	N.T.	N.T.	***N.T.***	N.T.	N.T.	N.T.
**Biome_G_202**	MAN	5.632552547405859	-53.66091898281266	*Mansonia*	***n.d.***		N.T.		N.T.	N.T.	N.T.	N.T.	***N.T.***	N.T.	N.T.	N.T.
**Biome_G_203**	MAN	5.632552547405859	-53.66091898281266	*Mansonia*	***n.d.***		N.T.		N.T.	N.T.	N.T.	N.T.	***N.T.***	N.T.	N.T.	N.T.
**Biome_G_204**	MAN	5.632552547405859	-53.66091898281266	*Mansonia*	***n.d.***		N.T.		N.T.	N.T.	N.T.	N.T.	***N.T.***	N.T.	N.T.	N.T.
**Biome_G_205**	MAN	5.632552547405859	-53.66091898281266	*Mansonia*	***n.d.***		N.T.		N.T.	N.T.	n.d.	N.T.	***N.T.***	N.T.	N.T.	N.T.
**Biome_G_206**	MAN	5.632552547405859	-53.66091898281266	*Mansonia*	***Ma. (Man.) titillans***	MT999303.1	*n.d.*		oui	*n.d.*	N.T.	n.d.	***n.d.***	*n.d.*	*n.d.*	*n.d.*
**Biome_G_207**	MAN	5.632552547405859	-53.66091898281266	*Mansonia*	***n.d.***		N.T.		N.T.	N.T.	N.T.	N.T.	***N.T.***	N.T.	N.T.	N.T.
**Biome_G_208**	LIB	4.915387042086516	-52.3613138909788	*Culex*	***n.d.***		N.T.		N.T.	N.T.	N.T.	N.T.	***N.T.***	N.T.	N.T.	N.T.
**Biome_G_209**	MAN	5.632552547405859	-53.66091898281266	*Aedes*	***n.d.***		N.T.		N.T.	N.T.	Pas de minion	N.T.	***N.T.***	N.T.	N.T.	N.T.
**Biome_G_210**	LIB	4.915387042086516	-52.3613138909788	*Culex*	***Cx. (Mel.) rabanicolus***	FGMOS2744-20	*Odocoileus virginianus cariacou*	KX381756.1	non	Pas de minion	Pas de minion	/	***Odocoileus cariacou***	Mammals	Artiodactyla	Doe
**Biome_G_211**	LIB	4.915387042086516	-52.3613138909788	*Culex*	***n.d.***		N.T.		N.T.	N.T.	N.T.	N.T.	***N.T.***	N.T.	N.T.	N.T.
**Biome_G_212**	LIB	4.915387042086516	-52.3613138909788	*Culex*	***n.d.***		N.T.		N.T.	N.T.	Pas de minion	N.T.	***N.T.***	N.T.	N.T.	N.T.
**Biome_G_213**	LIB	4.915387042086516	-52.3613138909788	*Mansonia*	***Ma. (Man.) titillans***	MT999303.1	*n.d.*		non	Pas de minion	Pas de minion	/	***n.d.***	*n.d.*	*n.d.*	*n.d.*
**Biome_G_214**	DDC	4.853576607619447	-52.26967869892846	*Culex*	***n.d.***		N.T.		N.T.	N.T.	N.T.	N.T.	***N.T.***	N.T.	N.T.	N.T.
**Biome_G_215**	DDC	4.853576607619447	-52.26967869892846	*Culex*	***n.d.***		N.T.		N.T.	N.T.	n.d.	N.T.	***N.T.***	N.T.	N.T.	N.T.
**Biome_G_216**	PAS	5.14781	-52.73996	*Coquillettidia*	***Cq. (Rhy.) venezuelensis***	OM630650.1	*Didelphis marsupialis*	KX381440.1	oui	*Didelphis marsupialis*	11523	oui	***Didelphis marsupialis***	*Mammals*	*Didelphimorphia*	*Marsupial*
**Biome_G_216**	PAS	5.14781	-52.73996	*Coquillettidia*	***Cq. (Rhy.) venezuelensis***	OM630650.1	*n.d.*		/	*Metachirus nudicaudatus*	3841	oui-m2	***Metachirus nudicaudatus***	Mammals	Didelphimorphia	Marsupial
**Biome_G_217**	ORG	5.55056	-53.46046	*Culex*	***Cx. (Mel.) pedroi***	GBCAB35634-24	*n.d.*		oui	*Dasyprocta leporina*	1560	non	*Dasyprocta leporina*	Mammals	Rodentia	Rodent
**Biome_G_218**	ORG	5.55056	-53.46046	*Culex*	***Cx. (Mel.) adamesi***	FGMOS2211-20	*n.d.*		oui	*n.d.*	n.d.	n.d.	***n.d.***	*n.d.*	*n.d.*	*n.d.*
**Biome_G_219**	ORG	5.55056	-53.46046	*Culex*	***Cx. (Mel.) portesi***	FGMOS2416-20	*Nyctanassa violacea*	MW524627.1	oui	*Nyctanassa violacea*	1644	non	***Nyctanassa violacea***	Birds	Pelecaniformes	Wild bird
**Biome_G_220**	ORG	5.55056	-53.46046	*Coquillettidia*	***Cq. (Rhy.) venezuelensis***	OM630650.1	*Canis lupus familiaris*	OQ341164.1	oui	*Canis lupus familiaris*	32958	non	***Canis lupus familiaris***	Mammals	Carnivora	Dog
**Biome_G_221**	ORG	5.55056	-53.46046	*Culex*	***n.d.***		N.T.		N.T.	N.T.	N.T.	N.T.	***N.T.***	N.T.	N.T.	N.T.
**Biome_G_222**	ORG	5.55056	-53.46046	*Culex*	***Cx. (Mel.) eastor***	FGMOS2695-20	*n.d.*		oui	*n.d.*	n.d.	n.d.	***n.d.***	*n.d.*	*n.d.*	*n.d.*
**Biome_G_223**	ORG	5.55056	-53.46046	*Culex*	***Cx. (Mel.) erraticus***	FGMOS3111-23	*n.d.*		oui	*n.d.*	n.d.	n.d.	***n.d.***	*n.d.*	*n.d.*	*n.d.*
**Biome_G_224**	ORG	5.55056	-53.46046	*Coquillettidia*	***Cq. (Rhy.) venezuelensis***	OM630650.1	*n.d.*		non	Pas de minion	Pas de minion	/	***n.d.***	*n.d.*	*n.d.*	*n.d.*
**Biome_G_225**	ORG	5.55056	-53.46046	*Culex*	***Cx. (Mel.) rabanicolus***	FGMOS2744-20	*n.d.*		non	Pas de minion	Pas de minion	/	***n.d.***	*n.d.*	*n.d.*	*n.d.*
**Biome_G_226**	ORG	5.55056	-53.46046	*Coquillettidia*	***Cq. (Rhy.) venezuelensis***	OP785627.1	*n.d.*		non	Pas de minion	Pas de minion	/	***n.d.***	*n.d.*	*n.d.*	*n.d.*
**Biome_G_227**	ORG	5.55056	-53.46046	*Coquillettidia*	***Cq. (Rhy.) venezuelensis***	OM630650.1	*n.d.*		non	Pas de minion	Pas de minion	/	***n.d.***	*n.d.*	*n.d.*	*n.d.*
**Biome_G_228**	ORG	5.55056	-53.46046	*Coquillettidia*	***Cq. (Rhy.) venezuelensis***	OM630650.1	*n.d.*		non	Pas de minion	Pas de minion	/	***n.d.***	*n.d.*	*n.d.*	*n.d.*
**Biome_G_229**	ORG	5.55056	-53.46046	*Culex*	***Cx. (Ads.) amazonensis***	FGMOS2756-20	*n.d.*		non	Pas de minion	Pas de minion	/	***n.d.***	*n.d.*	*n.d.*	*n.d.*
**Biome_G_230**	ORG	5.55056	-53.46046	*Culex*	***Cx. (Ads.) amazonensis***	FGMOS2756-20	*n.d.*		non	Pas de minion	Pas de minion	/	***n.d.***	*n.d.*	*n.d.*	*n.d.*
**Biome_G_231**	ORG	5.55056	-53.46046	*Culex*	***Cx. (Mel.) rabanicolus***	FGMOS2744-20	*n.d.*		non	Pas de minion	Pas de minion	/	***n.d.***	*n.d.*	*n.d.*	*n.d.*
**Biome_G_232**	ORG	5.55056	-53.46046	*Culex*	***Cx. (Mel.) adamesi***	FGMOS2220-20	*n.d.*		oui	*n.d.*	n.d.	n.d.	***n.d.***	*n.d.*	*n.d.*	*n.d.*
**Biome_G_233**	ORG	5.55056	-53.46046	*Culex*	***Cx. (Mel.) rabanicolus***	FGMOS2744-20	*n.d.*		oui	*n.d.*	n.d.	n.d.	***n.d.***	*n.d.*	*n.d.*	*n.d.*
**Biome_G_234**	ORG	5.55056	-53.46046	*Culex*	***Cx. (Mel.) vaxus***	FGMOS1713-20	*n.d.*		oui	*n.d.*	n.d.	n.d.	***n.d.***	*n.d.*	*n.d.*	*n.d.*
**Biome_G_235**	COU	5.457620389016933	-53.17713266584141	*Coquillettidia*	***Cq. (Rhy.) albicosta***	OP785646.1	*n.d.*		oui	*n.d.*	n.d.	n.d.	***n.d.***	*n.d.*	*n.d.*	*n.d.*
**Biome_G_236**	COU	5.457620389016933	-53.17713266584141	*Coquillettidia*	***Cq. (Rhy.) albicosta***	OP785646.1	*n.d.*		oui	*n.d.*	n.d.	n.d.	***n.d.***	*n.d.*	*n.d.*	*n.d.*
**Biome_G_237**	COU	5.457620389016933	-53.17713266584141	*Coquillettidia*	***Cq. (Rhy.) albicosta***	OP785646.1	*n.d.*		oui	*n.d.*	n.d.	n.d.	***n.d.***	*n.d.*	*n.d.*	*n.d.*
**Biome_G_238**	VIG	5.270323	-52.961293	*Culex*	***Cx. (Mel.) inadmirabilis***	FGMOS3035-22	*n.d.*		oui	*n.d.*	n.d.	n.d.	***n.d.***	*n.d.*	*n.d.*	*n.d.*
**Biome_G_239**	PAL	4.051826178135371	-51.66942020699708	*Culex*	***Cx. (Cux.) quinquefasciatus***	FGMOS2276-20	*n.d.*		oui	*Canis lupus familiaris*	240	non	***Canis lupus familiaris***	Mammals	Carnivora	Dog
**Biome_G_240**	PAL	4.051826178135371	-51.66942020699708	*Culex*	***Cx. (Cux.) quinquefasciatus***	FGMOS2276-20	*n.d.*		oui	*n.d.*	n.d.	n.d.	***n.d.***	*n.d.*	*n.d.*	*n.d.*
**Biome_G_241**	PAL	4.051826178135371	-51.66942020699708	*Culex*	***Cx. (Cux.) quinquefasciatus***	FGMOS2276-20	*n.d.*		oui	*n.d.*	n.d.	n.d.	***n.d.***	*n.d.*	*n.d.*	*n.d.*
**Biome_G_242**	PAL	4.051826178135371	-51.66942020699708	*Culex*	***Cx. (Cux.) quinquefasciatus***	FGMOS2276-20	*n.d.*		oui	*n.d.*	n.d.	n.d.	***n.d.***	*n.d.*	*n.d.*	*n.d.*
**Biome_G_243**	PAL	4.051826178135371	-51.66942020699708	*Culex*	***Cx. (Cux.) quinquefasciatus***	FGMOS2276-20	*n.d.*		oui	*n.d.*	n.d.	n.d.	***n.d.***	*n.d.*	*n.d.*	*n.d.*
**Biome_G_244**	PAL	4.051826178135371	-51.66942020699708	*Culex*	***Cx. (Cux.) quinquefasciatus***	FGMOS2276-20	*n.d.*		oui	*n.d.*	n.d.	n.d.	***n.d.***	*n.d.*	*n.d.*	*n.d.*
**Biome_G_245**	PAL	4.051826178135371	-51.66942020699708	*Culex*	***Cx. (Cux.) quinquefasciatus***	FGMOS2276-20	*n.d.*		oui	*n.d.*	n.d.	n.d.	***n.d.***	*n.d.*	*n.d.*	*n.d.*
**Biome_G_246**	PAL	4.051826178135371	-51.66942020699708	*Culex*	***Cx. (Cux.) quinquefasciatus***	FGMOS2276-20	*n.d.*		oui	*n.d.*	n.d.	n.d.	***n.d.***	*n.d.*	*n.d.*	*n.d.*
**Biome_G_247**	PAL	4.051826178135371	-51.66942020699708	*Culex*	***Cx. (Cux.) quinquefasciatus***	FGMOS2276-20	*Canis lupus familiaris*	OQ341164.1	oui	*Canis lupus familiaris*	21843	non	***Canis lupus familiaris***	Mammals	Carnivora	Dog

**Biome_G_248**	PAL	4.051826178135371	-51.66942020699708	*Culex*	***Cx. (Cux.) quinquefasciatus***	FGMOS2276-20	*n.d.*		oui	*n.d.*	n.d.	n.d.	***n.d.***	*n.d.*	*n.d.*	*n.d.*
**Biome_G_249**	PAL	4.051826178135371	-51.66942020699708	*Culex*	***Cx. (Cux.) quinquefasciatus***	FGMOS2276-20	*n.d.*		oui	*n.d.*	n.d.	n.d.	***n.d.***	*n.d.*	*n.d.*	*n.d.*
**Biome_G_250**	ORG	5.55056	-53.46046	*Culex*	***n.d.***		N.T.		N.T.	N.T.	N.T.	N.T.	***N.T.***	N.T.	N.T.	N.T.
**Biome_G_251**	ORG	5.55056	-53.46046	*Culex*	***Cx. (Mel.) eastor***	FGMOS2695-20	*n.d.*		oui	*Homo sapiens*	17870	non	***Homo sapiens***	Mammals	Primates	Human
**Biome_G_252**	ORG	5.55056	-53.46046	*Culex*	***Cx. (Mel.) phlogistus***	FGMOS3175-23	*n.d.*		oui	*n.d.*	n.d.	n.d.	***n.d.***	*n.d.*	*n.d.*	*n.d.*
**Biome_G_253**	COU	5.457620389016933	-53.17713266584141	*Aedeomyia*	***Ad. (Ady.) squamipennis***	MW110470.1	*n.d.*		oui	*n.d.*	n.d.	n.d.	***n.d.***	*n.d.*	*n.d.*	*n.d.*
**Biome_G_254**	VIG	5.270323	-52.961293	*Culex*	***n.d.***		N.T.		N.T.	N.T.	N.T.	N.T.	***N.T.***	N.T.	N.T.	N.T.
**Biome_G_255**	COU	5.457620389016933	-53.17713266584141	*Aedeomyia*	***n.d.***		N.T.		N.T.	N.T.	N.T.	N.T.	***N.T.***	N.T.	N.T.	N.T.
**Biome_G_256**	COU	5.457620389016933	-53.17713266584141	*Aedeomyia*	***n.d.***		N.T.		N.T.	N.T.	N.T.	N.T.	***N.T.***	N.T.	N.T.	N.T.
**Biome_G_257**	OUA	4.209156314357502	-51.67445725150719	*Culex*	***Cx. (Mel.) portesi***	FGMOS2416-20	*n.d.*		oui	*Hylaeamys yunganus*	22536	non	***Hylaeamys yunganus***	Mammals	Rodentia	Rodent
**Biome_G_258**	OUA	4.209156314357502	-51.67445725150719	*Culex*	***Cx. (Mel.) portesi***	FGMOS2416-20	*n.d.*		oui	*n.d.*	n.d.	n.d.	***n.d.***	*n.d.*	*n.d.*	*n.d.*
**Biome_G_259**	OUA	4.209156314357502	-51.67445725150719	*Culex*	***Cx. (Mel.) portesi***	FGMOS2416-20	*n.d.*		oui	*Homo sapiens*	54070	non	***Homo sapiens***	Mammals	Primates	Human
**Biome_G_260**	OUA	4.209156314357502	-51.67445725150719	*Culex*	***Cx. (Mel.) portesi***	FGMOS2416-20	*n.d.*		oui	*n.d.*	n.d.	n.d.	***n.d.***	*n.d.*	*n.d.*	*n.d.*
**Biome_G_261**	OUA	4.209156314357502	-51.67445725150719	*Culex*	***Cx. (Mel.) portesi***	FGMOS2416-20	*n.d.*		oui	*Gallus gallus*	2510	non	***Gallus gallus***	Birds	Galliformes	Domestic bird
**Biome_G_262**	OUA	4.209156314357502	-51.67445725150719	*Culex*	***Cx. (Mel.) portesi***	FGMOS2416-20	*n.d.*		oui	*Hylaeamys yunganus*	1146	non	***Hylaeamys yunganus***	Mammals	Rodentia	Rodent
**Biome_G_263**	OUA	4.209156314357502	-51.67445725150719	*Culex*	***Cx. (Mel.) portesi***	FGMOS2416-20	*n.d.*		oui	*Nectomys rattus*	1274	non	***Nectomys rattus***	Mammals	Rodentia	Rodent
**Biome_G_264**	OUA	4.209156314357502	-51.67445725150719	*Culex*	***Cx. (Mel.) portesi***	FGMOS2416-20	*n.d.*		oui	*Sapajus apella*	107140	non	***Sapajus apella***	Mammals	Primates	Non-human primate
**Biome_G_265**	OUA	4.209156314357502	-51.67445725150719	*Culex*	***Cx. (Mel.) portesi***	FGMOS2416-20	*n.d.*		oui	*Nectomys rattus*	2886	non	***Nectomys rattus***	Mammals	Rodentia	Rodent
**Biome_G_266**	OUA	4.209156314357502	-51.67445725150719	*Uranotaenia*	***n.d.***		N.T.		N.T.	N.T.	N.T.	N.T.	***N.T.***	N.T.	N.T.	N.T.
**Biome_G_267**	PAL	4.051826178135371	-51.66942020699708	*Culex*	***Cx. (Mel.) portesi***	FGMOS2416-20	*Philander opossum*	KX381605.1	oui	*Philander opossum*	10648	non	***Philander opossum***	Mammals	Didelphimorphia	Marsupial
**Biome_G_268**	PAL	4.051826178135371	-51.66942020699708	*Culex*	***Cx. (Mel.) portesi***	FGMOS2416-20	*Marmosa murina*	KX381433.1	oui	*Marmosa murina*	117	non	***Marmosa murina***	Mammals	Didelphimorphia	Marsupial
**Biome_G_269**	PAL	4.051826178135371	-51.66942020699708	*Culex*	***Cx. (Mel.) portesi***	FGMOS2416-20	*Marmosa murina*	KX381433.1	oui	*Marmosa murina*	4097	non	***Marmosa murina***	Mammals	Didelphimorphia	Marsupial
**Biome_G_270**	PAL	4.051826178135371	-51.66942020699708	*Culex*	***Cx. (Mel.) vomerifer***	FGMOS2191-20	*Philander opossum*	KX381605.1	oui	*Philander opossum*	4178	non	***Philander opossum***	Mammals	Didelphimorphia	Marsupial
**Biome_G_271**	PAL	4.051826178135371	-51.66942020699708	*Uranotaenia*	***n.d.***		N.T.		N.T.	N.T.	N.T.	N.T.	***N.T.***	N.T.	N.T.	N.T.
**Biome_G_272**	PAL	4.051826178135371	-51.66942020699708	*Uranotaenia*	***n.d.***		N.T.		N.T.	N.T.	N.T.	N.T.	***N.T.***	N.T.	N.T.	N.T.
**Biome_G_273**	PAL	4.051826178135371	-51.66942020699708	*Culex*	***Cx. (Mel.) portesi***	FGMOS2416-20	*Didelphis marsupialis*	KX381440.1	oui	*Didelphis marsupialis*	2688	oui	***Didelphis marsupialis***	Mammals	Didelphimorphia	Marsupial
**Biome_G_273**	PAL	4.051826178135371	-51.66942020699708	*Culex*	***Cx. (Mel.) portesi***	FGMOS2416-20	*n.d.*		/	*Metachirus nudicaudatus*	896	oui-m2	***Metachirus nudicaudatus***	Mammals	Didelphimorphia	Marsupial
**Biome_G_274**	PAL	4.051826178135371	-51.66942020699708	*Culex*	***Cx. (Mel.) portesi***	FGMOS2416-20	*n.d.*		oui	*n.d.*	n.d.	n.d.	***n.d.***	*n.d.*	*n.d.*	*n.d.*
**Biome_G_275**	PAL	4.051826178135371	-51.66942020699708	*Culex*	***Cx. (Mel.) dunni***	FGMOS2750-20	*n.d.*		oui	*n.d.*	n.d.	n.d.	***n.d.***	*n.d.*	*n.d.*	*n.d.*
**Biome_G_276**	PAL	4.051826178135371	-51.66942020699708	*Culex*	***Cx. (Mel.) contei***	FGMOS2767-20	*n.d.*		oui	*n.d.*	n.d.	n.d.	***n.d.***	*n.d.*	*n.d.*	*n.d.*
**Biome_G_277**	PAL	4.051826178135371	-51.66942020699708	*Uranotaenia*	***n.d.***		N.T.		N.T.	N.T.	N.T.	N.T.	***N.T.***	N.T.	N.T.	N.T.
**Biome_G_278**	PAL	4.051826178135371	-51.66942020699708	*Uranotaenia*	***n.d.***		N.T.		N.T.	N.T.	N.T.	N.T.	***N.T.***	N.T.	N.T.	N.T.
**Biome_G_279**	PAL	4.051826178135371	-51.66942020699708	*Uranotaenia*	***n.d.***		N.T.		N.T.	N.T.	N.T.	N.T.	***N.T.***	N.T.	N.T.	N.T.
**Biome_G_280**	PAL	4.051826178135371	-51.66942020699708	*Uranotaenia*	***n.d.***		N.T.		N.T.	N.T.	N.T.	N.T.	***N.T.***	N.T.	N.T.	N.T.
**Biome_G_281**	PAL	4.051826178135371	-51.66942020699708	*Uranotaenia*	***n.d.***		N.T.		N.T.	N.T.	N.T.	N.T.	***N.T.***	N.T.	N.T.	N.T.
**Biome_G_282**	MAT	4.8276386873942805	-52.31655054746172	*Anopheles*	***An. darlingi***	MH924508.1	*n.d.*		non	Pas de minion	Pas de minion	/	***n.d.***	*n.d.*	*n.d.*	*n.d.*
**Biome_G_283**	MAT	4.8276386873942805	-52.31655054746172	*Anopheles*	***An. darlingi***	MH924552.1	*Homo sapiens*	MF437201.1	non	Pas de minion	Pas de minion	/	***Homo sapiens***	Mammals	Primates	Human
**Biome_G_284**	MAT	4.8276386873942805	-52.31655054746172	*Anopheles*	***An. darlingi***	JF923695.1	*Homo sapiens*	MF437201.1	non	Pas de minion	Pas de minion	/	***Homo sapiens***	Mammals	Primates	Human
**Biome_G_285**	MAT	4.8276386873942805	-52.31655054746172	*Coquillettidia*	***n.d.***		N.T.		N.T.	N.T.	N.T.	N.T.	***N.T.***	N.T.	N.T.	N.T.
**Biome_G_286**	MAT	4.8276386873942805	-52.31655054746172	*Coquillettidia*	***n.d.***		N.T.		N.T.	N.T.	N.T.	N.T.	***N.T.***	N.T.	N.T.	N.T.
**Biome_G_287**	MAT	4.8276386873942805	-52.31655054746172	*Coquillettidia*	***n.d.***		N.T.		N.T.	N.T.	N.T.	N.T.	***N.T.***	N.T.	N.T.	N.T.
**Biome_G_288**	MAT	4.8276386873942805	-52.31655054746172	*Culex*	***Cx. (Mel.) dunni***	KX779797.1	*Homo sapiens*	MF437201.1	non	Pas de minion	Pas de minion	/	***Homo sapiens***	Mammals	Primates	Human
**Biome_G_289**	MAT	4.8276386873942805	-52.31655054746172	*Culex*	***Cx. (Cux.) quinquefasciatus***	FGMOS2276-20	*Canis lupus familiaris*	OM743426.1	non	Pas de minion	Pas de minion	/	***Canis lupus familiaris***	Mammals	Carnivora	Dog
**Biome_G_290**	MAT	4.8276386873942805	-52.31655054746172	*Anopheles*	***An. darlingi***	JF923695.1	*Homo sapiens*	MF437201.1	non	Pas de minion	Pas de minion	/	***Homo sapiens***	Mammals	Primates	Human
**Biome_G_291**	MAT	4.8276386873942805	-52.31655054746172	*Coquillettidia*	***Cq. (Rhy.) albicosta***	OP785646.1	*n.d.*		non	Pas de minion	Pas de minion	/	***n.d.***	*n.d.*	*n.d.*	*n.d.*
**Biome_G_292**	MAT	4.8276386873942805	-52.31655054746172	*Coquillettidia*	***Cq. (Rhy.) albicosta***	OP785646.1	*Dasyprocta leporina*	KX381742.1	non	Pas de minion	Pas de minion	/	*Dasyprocta leporina*	Mammals	Rodentia	Rodent
**Biome_G_293**	MAT	4.8276386873942805	-52.31655054746172	*Coquillettidia*	***Cq. (Rhy.) venezuelensis***	OM630650.1	*Didelphis marsupialis*	KX381440.1	non	Pas de minion	Pas de minion	/	***Didelphis marsupialis***	Mammals	Didelphimorphia	Marsupial
**Biome_G_294**	MAT	4.8276386873942805	-52.31655054746172	*Coquillettidia*	***Cq. (Rhy.) venezuelensis***	OM630651.1	*Canis lupus familiaris*	OR129838.1	non	Pas de minion	Pas de minion	/	***Canis lupus familiaris***	Mammals	Carnivora	Dog
**Biome_G_295**	MAT	4.8276386873942805	-52.31655054746172	*Coquillettidia*	***Cq. (Rhy.) venezuelensis***	OM630650.1	*Didelphis marsupialis*	KX381440.1	non	Pas de minion	Pas de minion	/	***Didelphis marsupialis***	Mammals	Didelphimorphia	Marsupial
**Biome_G_296**	MAT	4.8276386873942805	-52.31655054746172	*Coquillettidia*	***Cq. (Rhy.) venezuelensis***	OM630651.1	*Didelphis marsupialis*	KX381440.1	non	Pas de minion	Pas de minion	/	***Didelphis marsupialis***	Mammals	Didelphimorphia	Marsupial
**Biome_G_297**	MAT	4.8276386873942805	-52.31655054746172	*Coquillettidia*	***Cq. (Rhy.) venezuelensis***	OM630651.1	*Didelphis marsupialis*	KX381440.1	non	Pas de minion	Pas de minion	/	***Didelphis marsupialis***	Mammals	Didelphimorphia	Marsupial
**Biome_G_298**	MAT	4.8276386873942805	-52.31655054746172	*Coquillettidia*	***Cq. (Rhy.) venezuelensis***	OM630650.1	*n.d.*		non	Pas de minion	Pas de minion	/	***n.d.***	*n.d.*	*n.d.*	*n.d.*
**Biome_G_299**	MAT	4.8276386873942805	-52.31655054746172	*Coquillettidia*	***Cq. (Rhy.) venezuelensis***	OM630650.1	*n.d.*		non	Pas de minion	Pas de minion	/	***n.d.***	*n.d.*	*n.d.*	*n.d.*
**Biome_G_300**	MAT	4.8276386873942805	-52.31655054746172	*Coquillettidia*	***Cq. (Rhy.) venezuelensis***	OM630650.1	*Myrmecophaga tridactyla*	MH142215.1	non	Pas de minion	Pas de minion	/	***Myrmecophaga tridactyla***	Mammals	Pilosa	anteater
**Biome_G_301**	MAT	4.8276386873942805	-52.31655054746172	*Coquillettidia*	***Cq. (Rhy.) venezuelensis***	OM630650.1	*Dasypus guianensis*	KX381527.1	non	Pas de minion	Pas de minion	/	***Dasypus guianensis***	Mammals	Cingulata	Armadillo
**Biome_G_302**	MAT	4.8276386873942805	-52.31655054746172	*Coquillettidia*	***Cq. (Rhy.) albicosta***	OP785646.1	*Dasyprocta leporina*	KX381742.1	non	Pas de minion	Pas de minion	/	*Dasyprocta leporina*	Mammals	Rodentia	Rodent
**Biome_G_303**	MAT	4.8276386873942805	-52.31655054746172	*Culex*	***Cx. (Cux.) quinquefasciatus***	FGMOS2276-20	*Homo sapiens*	MF437201.1	non	Pas de minion	Pas de minion	/	***Homo sapiens***	Mammals	Primates	Human
**Biome_G_304**	MAT	4.8276386873942805	-52.31655054746172	*Coquillettidia*	***Cq. (Rhy.) albicosta***	OP785646.1	*Canis lupus familiaris*	OR129838.1	non	Pas de minion	Pas de minion	/	***Canis lupus familiaris***	Mammals	Carnivora	Dog
**Biome_G_305**	MAT	4.8276386873942805	-52.31655054746172	*Coquillettidia*	***Cq. (Rhy.) venezuelensis***	OM630650.1	*Leopardus wiedii*	KX381694.1	non	Pas de minion	Pas de minion	/	***Leopardus wiedii***	Mammals	Carnivora	tiger cat
**Biome_G_306**	MAT	4.8276386873942805	-52.31655054746172	*Coquillettidia*	***Cq. (Rhy.) venezuelensis***	OM630651.1	*n.d.*		non	Pas de minion	Pas de minion	/	***n.d.***	*n.d.*	*n.d.*	*n.d.*
**Biome_G_307**	MAT	4.8276386873942805	-52.31655054746172	*Coquillettidia*	***Cq. (Rhy.) venezuelensis***	OM630651.1	*n.d.*		non	Pas de minion	Pas de minion	/	***n.d.***	*n.d.*	*n.d.*	*n.d.*
**Biome_G_308**	MAT	4.8276386873942805	-52.31655054746172	*Culex*	***Cx. (Mel.) rabelloi***	KX779859.1	*Makalata didelphoides*	KX381543.1	non	Pas de minion	Pas de minion	/	***Makalata didelphoides***	Mammals	Rodentia	Rodent
**Biome_G_309**	MAT	4.8276386873942805	-52.31655054746172	*Coquillettidia*	***Cq. (Rhy.) venezuelensis***	OM630651.1	*Dasyprocta leporina*	KX381742.1	non	Pas de minion	Pas de minion	/	*Dasyprocta leporina*	Mammals	Rodentia	Rodent
**Biome_G_310**	MAT	4.8276386873942805	-52.31655054746172	*Coquillettidia*	***n.d.***		N.T.		N.T.	N.T.	N.T.	N.T.	***N.T.***	N.T.	N.T.	N.T.
**Biome_G_311**	MAT	4.8276386873942805	-52.31655054746172	*Coquillettidia*	***n.d.***		N.T.		N.T.	N.T.	N.T.	N.T.	***N.T.***	N.T.	N.T.	N.T.
**Biome_G_312**	MAT	4.8276386873942805	-52.31655054746172	*Culex*	***Cx. (Mel.) portesi***	FGMOS2416-20	*Canis lupus familiaris*	OR129838.1	non	Pas de minion	Pas de minion	/	***Canis lupus familiaris***	Mammals	Carnivora	Dog
**Biome_G_313**	MAT	4.8276386873942805	-52.31655054746172	*Culex*	***n.d.***		N.T.		N.T.	N.T.	N.T.	N.T.	***N.T.***	N.T.	N.T.	N.T.

Species names in red indicate those found in common between Sanger and MinION sequencing

N.T. : Samples not tested

n.d. : Samples with no determination or with low identity (<97%)
